# PD-1 and PD-L1: architects of immune symphony and immunotherapy breakthroughs in cancer treatment

**DOI:** 10.3389/fimmu.2023.1296341

**Published:** 2023-12-01

**Authors:** Adil Parvez, Furqan Choudhary, Priyal Mudgal, Rahila Khan, Kamal A. Qureshi, Humaira Farooqi, Ashok Aspatwar

**Affiliations:** ^1^ Department of Biotechnology, School of Chemical and Life Sciences, Jamia, Hamdard, New Delhi, India; ^2^ Department of Pharmaceutics, Unaizah College of Pharmacy, Qassim University, Unaizah, Qassim, Saudi Arabia; ^3^ Faculty of Medicine and Health Technology, Tampere University, Tampere, Finland

**Keywords:** PD-1, PD-L1, cancer immunotherapy, immune checkpoint inhibitors, combination therapy, monoclonal antibody

## Abstract

PD-1 (Programmed Cell Death Protein-1) and PD-L1 (Programmed Cell Death Ligand-1) play a crucial role in regulating the immune system and preventing autoimmunity. Cancer cells can manipulate this system, allowing them to escape immune detection and promote tumor growth. Therapies targeting the PD-1/PD-L1 pathway have transformed cancer treatment and have demonstrated significant effectiveness against various cancer types. This study delves into the structure and signaling dynamics of PD-1 and its ligands PD-L1/PD-L2, the diverse PD-1/PD-L1 inhibitors and their efficacy, and the resistance observed in some patients. Furthermore, this study explored the challenges associated with the PD-1/PD-L1 inhibitor treatment approach. Recent advancements in the combination of immunotherapy with chemotherapy, radiation, and surgical procedures to enhance patient outcomes have also been highlighted. Overall, this study offers an in-depth overview of the significance of PD-1/PD-L1 in cancer immunotherapy and its future implications in oncology.

## Graphical Abstract

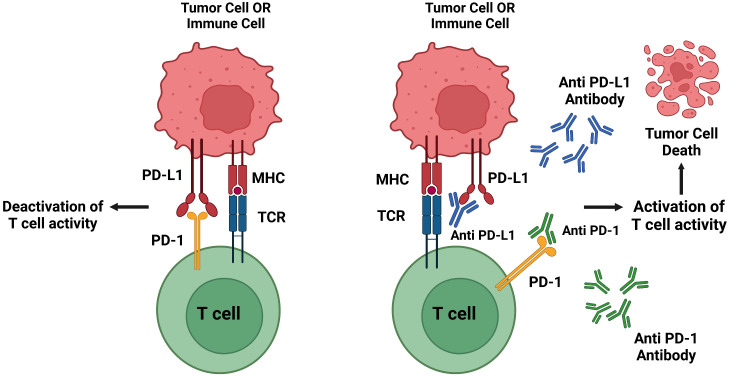

## 1 Introduction

Cancer is a significant public health concern that contributes to global mortality and morbidity rates. In 2020, it was reported that there were approximately 18.1 million new cases of cancer worldwide, excluding non-melanoma skin cancer, with 8.8 million (48%) in females and 9.3 million (52%) in males. This resulted in a ratio of 10 males to every 9.5 females. The global age-standardized incidence rate was 178.1 per 100,000 females and 206.9 per 100,000 males. The four most common types of cancer worldwide are breast, lung, bowel (including anus), and prostate cancers, which collectively account for 43% of all new cases (1). In 2023, it is projected that 1,958,310 new cancer cases and 609,820 cancer-related deaths will occur in the US. The incidence of prostate cancer increased by 3% annually from 2014 to 2019, resulting in 99,000 additional cases. However, lung cancer in women decreases at a slower pace than in men, and breast and uterine corpus cancers continue to increase (2). The incidence of liver cancer and melanoma stabilized in men aged ≥ 50 years and declined in younger men. A 65% drop in cervical cancer incidence among women in their early 20s who received the human papillomavirus vaccine foreshadows a reduction in cancers associated with the virus. Despite the pandemic, the cancer death rate has continued to decline in 2020 (by 1.5%), contributing to a 33% overall reduction since 1991 and an estimated 3.8 million deaths averted. Advancements in treatment, particularly for leukemia, melanoma, and kidney cancer, have led to rapid declines in mortality (2% annually from 2016 to 2020) despite the increasing incidence. Future progress may be hindered by the increasing incidence of breast, prostate, and uterine corpus cancers, which also have the largest racial disparities in mortality (2).

Despite recent advances in cancer therapies such as chemotherapy, radiation, and surgery, these methods frequently provide limited responses and substantial side effects. Immunotherapy has emerged as a viable method to overcome these limitations and improve cancer treatment outcomes (3). Immunotherapy is a recognized and powerful cancer treatment method that eradicates tumors by regulating anti-tumor immune responses (4). The immune system is essential for the detection and destruction of abnormal cells, including tumor cells. However, tumor cells can elude immune monitoring by developing immunological tolerance via many pathways, including the upregulation of immunological checkpoint molecules such as PD-1 and PD-L1 (5).

Immune checkpoint proteins control the start or stop of an immune response by being turned on or off, functioning as switches for immune function. PD-1 and its receptor, PD-L1, are essential immune checkpoint proteins that adversely control the equilibrium and function of T-cell immunological activity. T-cells are immune cells that identify and eliminate harmful or infected cells, such as cancer cells (6). PD-1 is found in T-cells, and PD-L1 is often found in cancer cells. The binding of PD-1 to its ligand PD-L1 may activate an inhibitory signal, resulting in decreased T-cell activity and anti-tumor immunity (7). Immune checkpoint inhibitors (ICIs) are a type of drug that blocks immune checkpoints, allowing the immune system to attack cancer cells. Monoclonal antibodies (mAbs) are among the major types of ICIs. Monoclonal antibodies are laboratory-produced molecules that target certain components of cancer or immune system cells, such as PD-1 and PD-L1. ICIs enhance effector T-cell function and provide long-term relief to patients with different malignancies (8).

Researchers have discovered that blocking the PD-1/PD-L1 pathway can reawaken cytotoxic T-cells and unleash the immune system against cancer cells. This method successfully cured various malignancies, including melanoma, non-small cell lung cancer, and bladder cancer (9).

The PD-1/PD-L1 pathway plays an integral role in facilitating tumor growth by evading the immune system. Blocking this pathway using checkpoint inhibitors has yielded notable therapeutic results in diverse cancer types. An in-depth understanding of this pathway is imperative in the contemporary landscape of advanced immunotherapy. This article offers an exhaustive analysis of the roles of PD-1 and PD-L1 in cancer immunology. This discourse will traverse the current body of knowledge on these proteins, with a particular emphasis on the clinical utility of PD-1/PD-L1 inhibitors in oncological treatments.

Moreover, this review underscores avenues for further research, addresses potential resistance modalities, explores the prospects of combined therapeutic strategies, and evaluates the long-term safety and effectiveness of PD-1/PD-L1 inhibitor therapies. Maintaining cognizance of these advancements is essential for the ongoing evolution of oncological practices. To further elucidate this pathway and its significance in oncology, let us begin by exploring the distinct roles of PD-1 and PD-L1.

## 2 PD-1 and PD-L1

Cancerous cells can be detected and eliminated by the immune system. However, cancer cells frequently evolve methods to elude immune system monitoring in their fight for survival. The PD-1/PD-L1 pathway controls the formation and maintenance of immunological tolerance in the microenvironment of a tumor. PD-1 and its ligands PD-L1 and PD-L2 govern the activation, proliferation, and release of cytotoxic substances from T-cells in cancer. With this foundational knowledge, we can further examine the specifics of PD-1 and PD-L1, their molecular structures, and critical roles in immune regulation.

### 2.1 PD-1

PD-1 (Pdcd1) was identified as a member of the immunoglobulin (lg) superfamily within the CD28/CTLA-4 family following its discovery in the early 1990s. PD-1 is an immunosuppressive receptor that is expressed during the immune reaction phase. PD-1 is a type I transmembrane protein with a molecular weight of approximately 50-55 kDa (10). On the surface of B-cells, T-cells, natural killer (NK) cells, and myeloid cells are the protein PD-1, which primarily regulates the actions of T-cells inside tissues and inhibits their potential to induce cell death in malignant situations. Dendritic cells (DCs) also express PD-1, which is activated by inflammatory stimuli, similar to T- and B-cells (6, 11).

**Figure 1:**
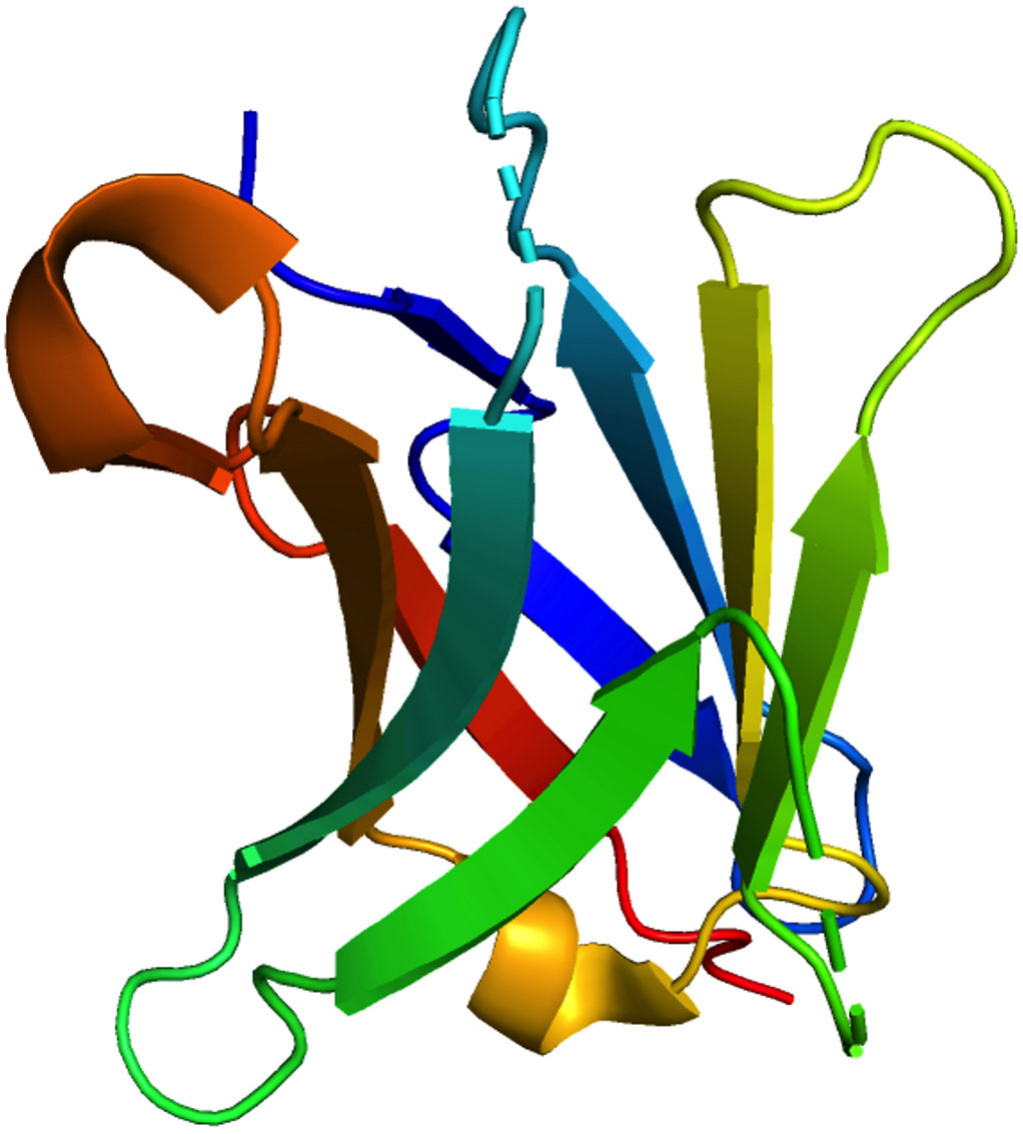
3D visualization of the crystal structure of PD-1 was performed using the PDB code 3RRQ. The various colors delineate distinct protein regions and conformations.

PD-1 exhibits a sequence similarity of 15% with CD28, 20% with CTLA4, and 13% with the induced T-cell co-stimulator at the amino acid level. PD-1 is composed of 288 amino acids, including an N-terminal IgV domain, transmembrane domain, intracellular cytoplasmic tail containing two tyrosine-based signaling motifs, and 20-amino acid linker connecting the IgV domain to the plasma domain. PD-1 recruits proteins with the N-terminal amino acid sequence VDYGEL and C-terminal amino acid sequence TEYATI. Both sequences combine to produce an immunoreceptor switch motif (ITSM) based on tyrosine, which is crucial for PD-1 inhibition (6, 12). The crystal structure of PD-1 is shown in **Figure 1**, accessed from the PDB database (PDB-3RRQ).

The *pdcd1* gene encodes the PD-1 protein, and various transcription factors facilitate its transcription. These include NFATc1, FoxO1, AP-1, STAT3, STAT4, ISGF3, NF-κB and IRF9 (13, 14). In CD4+ and CD8+ T-cells, macrophages, and HIV-specific T-cells, the expression of PD-1 is transcriptionally regulated by various factors, including Notch, CTCF, AhR, IRF9, T-bet, FoxO1, and the HIV-1 accessory protein Nef (15).

The CR-B and COR-C regions, which are conserved upstream regulatory elements, play a critical role in regulating PD-1 gene expression. In the CR-C region of TCD4 and TCD8, the binding site is associated with NFATc1 (NFAT2). C-Fos is also connected to the CR-B area, which increases PD-1 expression. When NFATc is active, it binds to the pdcd1 promoter, activating the gene. Furthermore, the synergistic effect of IFN-γ and IRF9 leads to enhanced PD-1 expression by binding to the promoter region of pdcd1, thereby contributing to T-cell dysfunction. Likewise, the release of tumor cells elicits the production of the AP-1 subunit c-FOS within cancer cells, subsequently amplifying the expression of PD-1 (13, 15). However, PD-1 may have both negative and positive effects, as it can be destructive and valuable. It functions as an immunosuppressive agent, modulates immunological tolerance, and exerts protective effects by reducing the regulation of undesirable immune responses. However, interference with the protective properties of the immune system may cause cancer cell development (13). While PD-1’s dual roles are intriguing, its ligand PD-L1 also plays a pivotal role in the intricate interplay of immune modulation. Let us delve more deeply into the properties and functions of PD-L1.

### 2.2 PD-L1 and PD-L2

**Figure 2:**
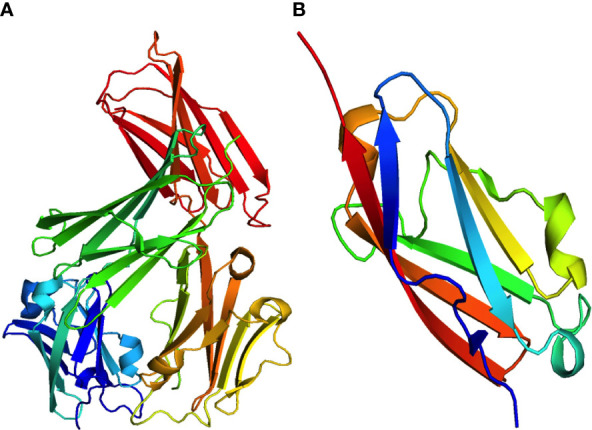
Crystal structures of two significant programmed cell death ligands, **(A)** PD-L1 (PDB:4Z18) and **(B)** PD-L2 (PDB:3BOV).

PD-1 exclusively interacts with programmed cell death ligands (PD-L1/PD-L2). Notably, PD-L1 is expressed in several cell types and organs, including hematopoietic cells (T-cells, B cells, macrophages, dendritic cells, and neutrophils), non-hematopoietic organs (heart, pancreas, placenta, vascular endothelium, muscle, liver, lung, eye, and skin tissues), and antigen-presenting cells (APC). In contrast, PD-L2 was detected in activated macrophages and dendritic cells. The engagement of PD-1 with its ligands inhibits T-cell activation signaling through the T-cell receptor (TCR) (11). Glycoprotein PD-L1, also known as B7-H1 or CD274, consists of 290 amino acids. It is a type I transmembrane protein that contains the IgC and IgV domains. Moreover, it is typically increased in several types of cancer cells or tumor stromal cells, which may be essential for avoiding host immune recognition (16). Interferon-gamma (IFN-γ) released by activated T-cells can upregulate the tumor cell surface protein PD-L1. The crystal structure of PD-L1 is shown in **Figure 2A**, accessed from the PDB database (PDB-4Z18).

Controlling PD-L1 expression has been the subject of much research. The regulation of PD-L1 expression involves an intricate network consisting of primary, non-immune-related, and secondary immune-related mechanisms. The primary factors contributing to the upregulation of PD-L1 can be attributed to a range of mechanisms, including (1) genomic abnormalities, (2) regulation by microRNAs, (3) activation of cancer-causing transcription factors and signaling pathways, and (4) post-translational modifications and trafficking. In contrast, secondary mechanisms primarily involve activating inflammatory signaling pathways triggered by soluble factors produced by immune cells within the tumor microenvironment (TME) (17).

The expression levels of PD-L1 are modulated through diverse mechanisms, which encompass genomic alterations such as amplification or translocation; epigenetic modifications such as methylation of histones or CpG islands; acetylation of histones; transcriptional control induced by inflammatory signals and oncogenic pathways; post-transcriptional regulation involving miRNAs, 3’-UTR status, RAS, and Angiotensin II; and post-translational modifications, including ubiquitination, phosphorylation, glycosylation, and palmitoylation (18).

The structure of PD-L1 can be categorized into three distinct domains: extracellular domain (ED), transmembrane domain (TM), and intracellular domain. The extracellular domain (ED) of PD-L1 comprises variable Ig regions, including distal and proximal segments. The intracellular domain of PD-L1 contains three conserved amino acid sequences, RMLD-VEKC, DTSSK, and QFEET. The RMLDVEKC motif plays a crucial role in facilitating the phosphorylation of signal transducer and activator of transcription 3 (STAT3), whereas the DTSSK motif impedes this phosphorylation process. Multiple signaling pathways and proteins regulate the expression of PD-L1 on cancerous cell surfaces. COX2/mPGES1/PGE2, hypoxia-inducible factor alpha (HIF1), nuclear factor-kappa-B p105 subunit (NF- κB), PI3K/AKT/mTOR, RAF/MEK/ERK/MAPK pathways, and STATs are among the proteins that undergo frequent mutations or upregulation throughout the malignant transformation process (18, 19).

In addition, PD-L2 is the second ligand for PD-1 and has a sequence similarity with PD-L1 of roughly 60% in humans. Compared to PD-L1, only activated dendritic cells (DCs), macrophages, and mast cells derived from the bone marrow, and B cells express PD-L2. Lipopolysaccharide (LPS) and B-cell receptors on B cells, as well as granulocyte-macrophage colony-stimulating factor (GM-CSF) and IL-4 on dendritic cells (DCs), can enhance the expression of PD-L2. While both PD-1 ligands are found in tumor cells and during chronic infections, the expression of PD-L2 is comparatively lower than that of PD-L1 (13, 18). The crystal structure of PD-L2 obtained from the PDB database (PDB-3BOV) is shown in **Figure 2B**.

PD-L2 inhibits T-cell activity in peripheral tissues. The role of PD-L2 in T-cell regulation has been the subject of debate and conflicting findings in the scientific literature. While specific studies have suggested that PD-L2 functions as a suppressive costimulatory molecule, others have proposed that it acts as a stimulatory molecule through a receptor distinct from PD-1. The interaction between PD-1 and PD-L2 strongly inhibits CD4+ T-cell TCR-mediated proliferation and cytokine production. Interactions between PD-L2 and PD-1 at low antigen concentrations inhibit powerful B7CD28 signals. At high antigen concentrations, interactions between PD-L2 and PD-1 inhibit cytokine production but have little effect on T-cell proliferation (20).

As we have delineated the structural intricacies and functional dynamics of PD-1, PD-L1, and PD-L2, it is crucial to understand the signaling processes that arise from their interactions, which serve as the foundation for numerous therapeutic interventions.

## 3 PD-1: PD-L1 signaling

The link between PD-1 and PD-L1 inhibits T-cell receptor signaling and functional activity. Suppression of T-cell activation and proliferation leads to a decrease in cytokine production and cytotoxic activity. Multiple studies have clarified the mechanism by which PD-1 and PD-L1 inhibit T-cell activation, and it has been shown that the overexpression of PD-L1 in cancer cells is associated with poor prognosis in a wide range of tumor types (21, 22).

**Figure 3:**
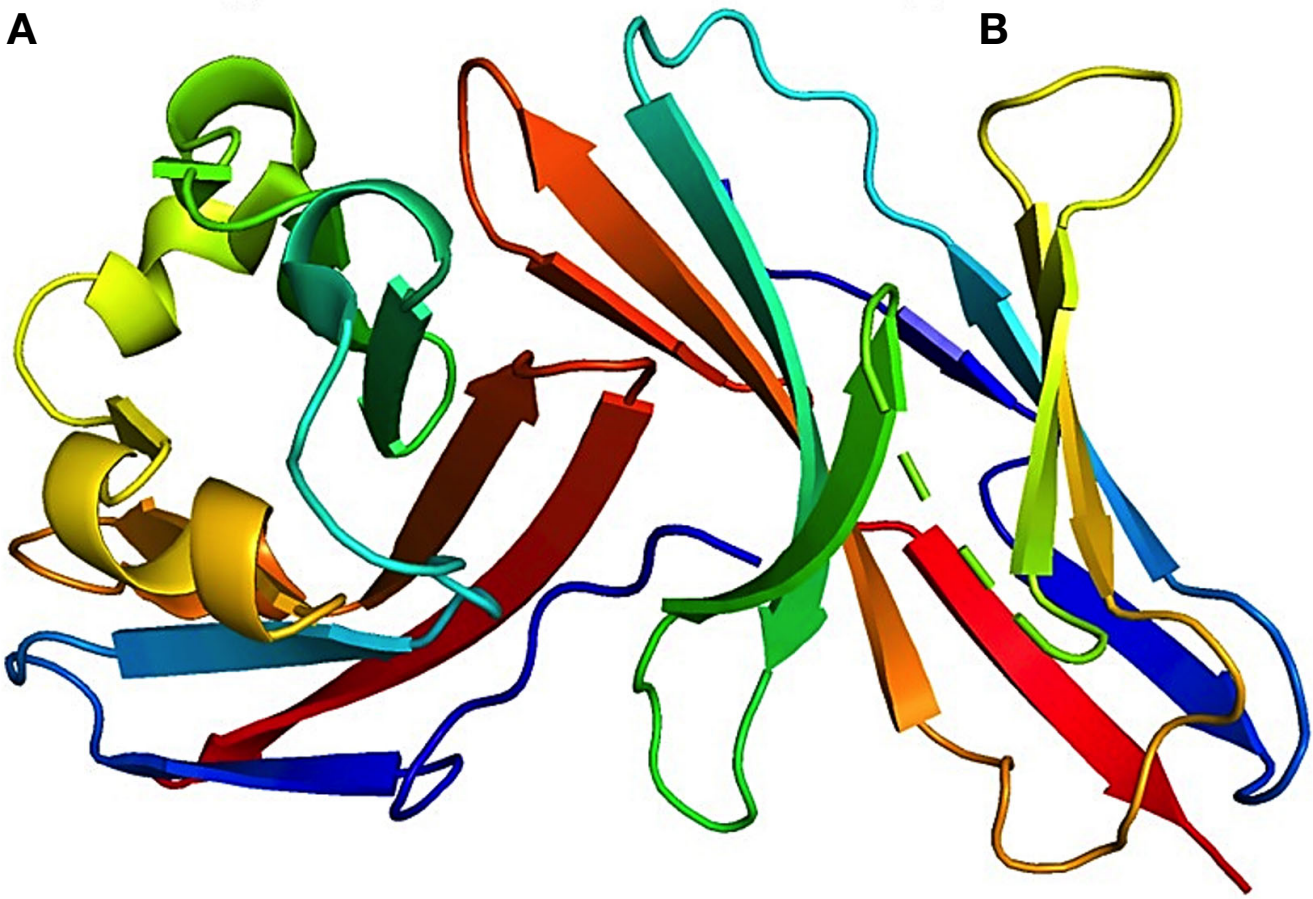
3D visualization of the crystal structure of the PD-1–PD-L1 complex. This image offers a detailed look at the structural alignment of the complex, highlighting that **(A)** represents PD-L1 chain and **(B)** represents PD-1 chain. The associated Protein Data Bank (PDB) reference for this structure is 4ZQK. This figure aids in the understanding of the intricate molecular interactions and conformation of the PD-1–PD-L1 interaction.

**Figure 4:**
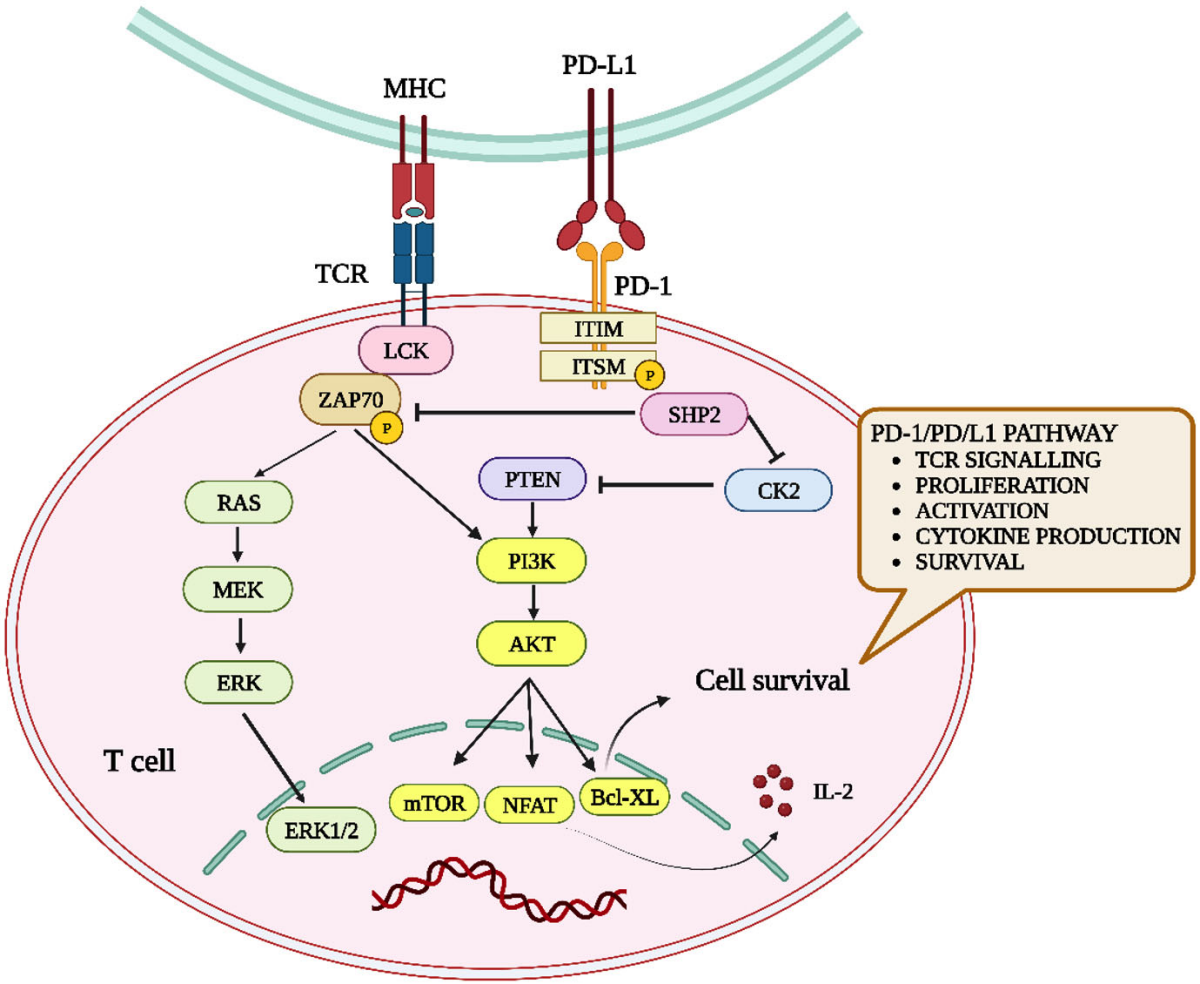
Overview of the impact of PD-1–PD-L1 interaction on T-cells. When PD-L1 binds to PD-1, it induces SHP-2, diminishing ZAP70 phosphorylation and dampening the RAS-MEK-ERK and PI3K-Akt pathways. This results in suppressed T-cell activity, including proliferation, activation, cytokine production, and longevity.

T-cell receptor (TCR) signaling activates tyrosine residues inside PD-1 when PD-1 binds to PD-L1. Protein tyrosine phosphatases are recruited by phosphorylated PD-1 (SHP1 and SHP2). When Src homology 2 (SH2) domain-containing protein tyrosine phosphatase (SHP2) is positioned near the T-cell receptor (TCR), it can reduce the phosphorylation of the 70-kDa zeta-associated protein (ZAP70) by tyrosine-protein kinase Lck (Lck). Consequently, this inhibitory action disrupts the subsequent signaling cascades of TCR. Furthermore, SHP2 directly repress casein kinase 2 (CK2) activity. PTEN, a serine-threonine phosphatase, functions as an inhibitor of the phosphoinositide 3-kinase and protein kinase B (PI3K-Akt) pathway upon PI3K activation. Conversely, CK2 typically inhibits PTEN phosphorylation. Upon inhibition of CK2 by SHP2, PTEN undergoes dephosphorylation, leading to subsequent inhibition of the PI3K-Akt pathway. SHP2 inhibits the RAS/MEK/ERK pathway by impeding RAS activation (16, 23). The interaction between both checkpoint proteins is shown in the crystal structure of the PD-1:PD-L1 complex (PDB-4ZQK) in **Figure 3**. The interaction between PD-1 and PD-L1 can potentially impact various downstream processes in T-cells. These actions involve regulation of the B-cell lymphoma-extra-large (Bcl-XL) pathway, influencing cellular survival and growth, along with the synthesis of IL-2 and Interferon (IFN). The mechanisms of the PD-1/PD-L1 pathway in T-cells are shown in **Figure 4**. Consequently, T-cells undergo various changes, including reduced proliferation, survival, cytokine generation, and other effector activities (16, 24).

In the tumor microenvironment (TME), the interaction between PD-1 and PD-L1 promotes immune surveillance mechanisms and facilitates cancer progression. Within the TME, the interaction of PD-L1 and PD-1 on activated T lymphocytes leads to impaired T-cell function and diminished proliferation, inhibition, and generation of IL-10. CD8+ T-cells expressing PD-1 and undergoing exhaustion exhibit diminished ability to produce cytolytic molecules and cytokines, including IL-2, IFN-γ, and tumor necrosis factor (TNF)-α. Consequently, these T-cells lose their effectiveness in eradicating cancerous cells (25).

Researchers have focused on the PD-1/PD-L1 pathway because of its intricate signaling and immunosuppressive roles. The manipulation of this pathway has shown significant clinical benefits in oncology. Herein, we explored therapeutic strategies targeting the PD-1/PD-L1 pathway, including recent advancements in PD-1/PD-L1 inhibitors.

## 4 Mechanisms and therapeutic applications of PD-1/PD-L1 inhibitors

The family of anti-cancer drugs known as PD-1/PD-L1 inhibitors suppresses the function of the immune checkpoint proteins PD-1 and PD-L1, which are present on cell membranes. By blocking the interaction between PD-1 and PD-L1, these inhibitors enable the immune system to eliminate tumors. Its anti-cancer benefits are also evident (7). These regulatory checkpoints are activated when immune checkpoint proteins, such as PD-1, present on T-cells, recognize and engage with corresponding counter proteins or ligands, such as PD-L1, expressed on neighboring cells, including potentially oncogenic cells. This interaction sends an inhibitory signal to T-cells, thereby reducing their ability to attack malignant cells. Immune checkpoint inhibitors block this interaction, disabling the inhibitory signal and empowering T-cells to effectively eliminate cancer cells (26).

Apart from focusing on inhibitors that target the PD-1/PD-L1 pathway, researchers have explored the potential of PD-L2 inhibitors as a promising approach to cancer immunotherapy. These drugs may suppress PD-1 protein on the surface of lymphocytes and PD-L1 and PD-L2 ligands generated by tumor cells. Despite its dynamic and diverse expression being a limiting feature, PD-L1 expression has been regarded as a predictive biomarker for cancer immunotherapy (26, 27).

Several PD-1/PD-L1 inhibitors are available, each with a unique molecular structure and a unique target profile. The following discussion focuses on several examples of these inhibitors:

### 4.1 Peptide-based PD-1/PD-L1 inhibitors

The first peptide-based inhibitor, AUNP-12, was patented in 2014. The peptide was designed to interfere with the PD-1–PD-L1 interaction, a critical checkpoint in the immune response. Inhibition of this interaction can potentially enhance the immune system’s ability to recognize and attack tumor cells. AUNP-12 is composed of 29 amino acids that form a branching structure. In animal studies, AUNP-12 reduced tumor cell proliferation and metastasis with few side effects. AUNP-12 has intrinsic advantages as an inhibitor or activator of protein-protein interactions when compared to small molecules and antibodies, making them promising therapeutic candidates. Multiple peptide-based inhibitors targeting the PD-1/PD-L1 interaction have been developed in addition to AUNP-12. A peptide with a similar 7-8 amino acid structure exhibited the most potent bioactivity in murine models of B16F10 melanoma cancer cells. Administration of this peptide resulted in a substantial reduction (64%) in lung metastases. The second compound was a cyclopeptide derivative consisting of 7-9 amino acids. Its cyclic structure is formed through an amide bond connecting the N- and C-termini of amino acid residues. Using crystal field stabilization energy (CFSE) detection, scientists observed that a cyclic peptide derivative could potentially enhance the proliferation of spleen cells in mice carrying human breast MDAMB-231 cancer cells, characterized by increased PD-L1 expression. Simultaneously, it reduced lung cancer progression by 54% in mice harboring melanoma B16F10 cells (28–30).

### 4.2 Small-molecule based PD-1/PD-L1 inhibitors

Recently, Bristol-Myers Squibb (BMS) has successfully developed a diverse collection of non-peptide small-molecule inhibitors specifically designed to target and block the PD-1/PD-L1 pathway. Among the identified compounds, BMS-8 and BMS-202 exhibited IC_50_ values of 146 nM and 18 nM, respectively. These inhibitors induce dimerization of PD-L1, leading to a decrease in the activation of PD-1. Additionally, compounds developed by BMS possess structural features that enable direct binding to PD-L1, thereby blocking the connection between PD-1 and PD-L1. Notably, LH1306 and LH1307 demonstrated IC_50_ values of 25 nM and 30 nM, respectively, indicating their potency in inhibiting the PD-1/PD-L1 pathway (31–33).

Furthermore, these inhibitors can impede PD-1 signal transduction during co-culture experiments by disrupting protein interactions between PD-1 and PD-L1. Small molecules can penetrate tissues more easily and can access intracellular targets. They can be designed to target PD-1/PD-L1 interaction and have therapeutic benefits. However, tumors may develop resistance faster, and they can be rapidly metabolized and excreted, reducing their efficacy (31–33).

In addition to directly inhibiting the PD-1/PD-L1 pathway, small molecule inhibitors exhibit synergy when paired with PD-1/PD-L1 inhibitors, particularly when targeting the epigenetic or metabolic pathways of specific immune cells. Research reveals that small compounds like EZH2 or IDO1 inhibitors might increase the efficacy of PD-1/PD-L1 inhibition, suggesting a viable combinatorial cancer immunotherapy pathway. According to reports, small compounds such as EZH2 inhibitors that disrupt epigenetic regulation or IDO1 inhibitors that modulate metabolic pathways have boosted the anti-tumor immunity induced by PD-1/PD-L1 inhibitors. This combinatorial method has the potential to overcome the limitations of monotherapy by delivering a more powerful anti-tumor immune response (34).

These findings highlight the potential of small molecule inhibitors not only as direct PD-1/PD-L1 antagonists but also as synergistic agents that modulate the immunological milieu, hence boosting the effectiveness of PD-1/PD-L1 inhibitors. This dual strategy, which targets the interplay between PD-1/PD-L1 and epigenetic or metabolic pathways, may broaden the treatment scope and enhance response rates in a larger patient population.

### 4.3 Antibody-based PD-1/PD-L1 inhibitors

Currently, there are 5,683 clinical studies evaluating anti-PD-1/PD-L1 mAbs as standalone treatments or in conjunction with other therapeutic approaches, with 4,897 active trials. Over the past five years, the total number of clinical studies has increased by 278 percent compared to the study conducted in 2017. Even though the overall number of clinical trials continues to rise annually, study data over the past few years show that the rate of increase has slowed. The overall number of clinical studies evaluating anti-PD-1/PD-L1 mAbs increased by 29 percent in the last year compared to a 50 percent increase from 2017 to 2018. In the analysis of FDA-approved PD-1/PD-L1-targeting mAbs and their comparison with other mAbs with the same target, it is evident that a considerable proportion (29 percent) of ongoing clinical studies investigating anti-PD-1/PD-L1 mAbs that are still in the developmental phase and have not yet received FDA approval exhibit significant effectiveness (35).

**Figure 5:**
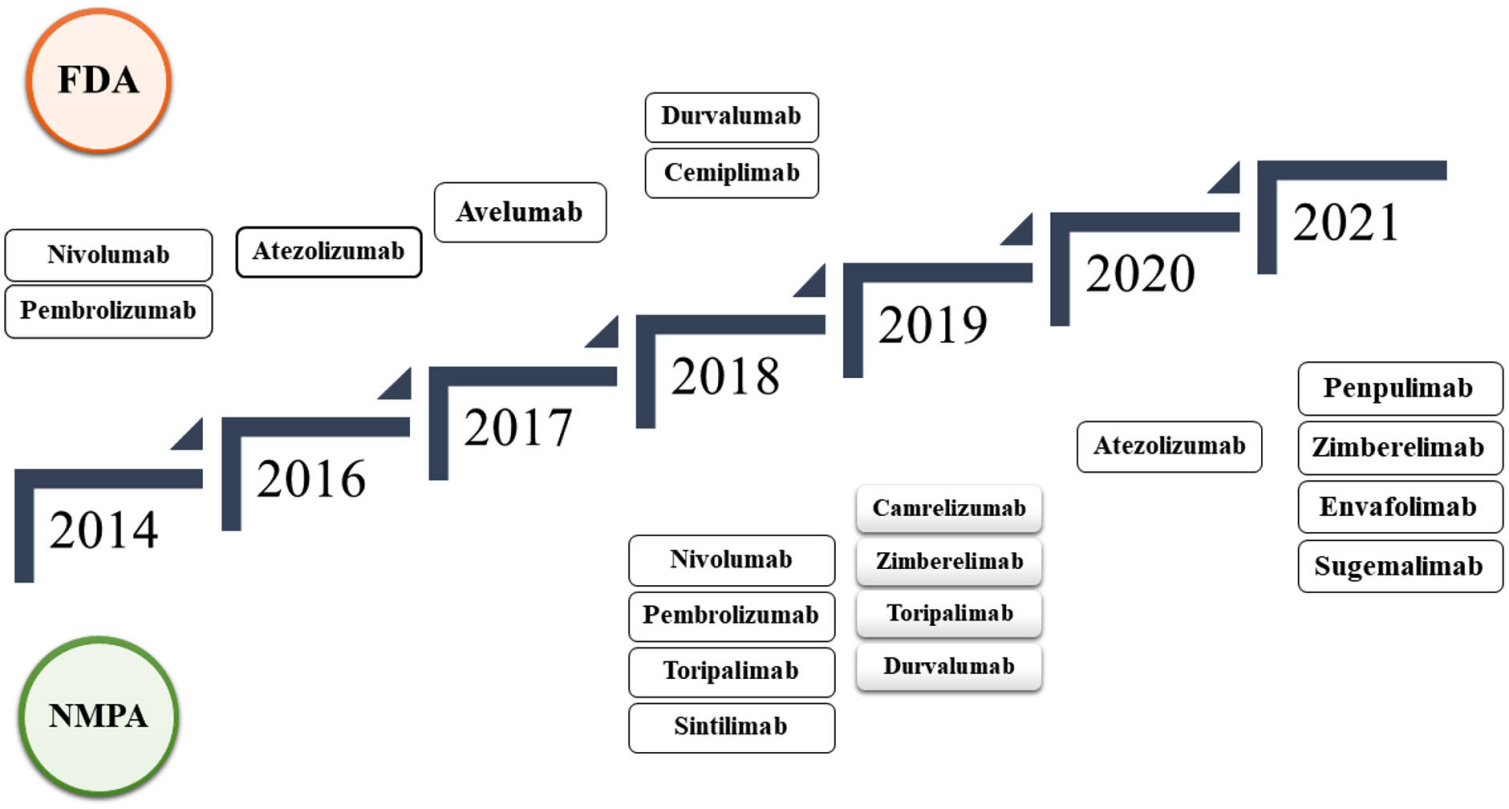
Timeline of FDA and NMPA-approved PD-1/PD-L1 immune checkpoint inhibitors from 2014 to 2021, detailing the progression of approvals over the years.

Sixteen PD-1/PD-L1 immune checkpoint inhibitors (ICIs) have shown outstanding effectiveness in various tumor types in the past few years, as shown in **Figure 5**. Since the release of nivolumab (36), the world’s first PD-1 inhibitor, in 2014, 10 PD-1 inhibitors have been developed pembrolizumab (37), cemiplimab (38), toripalimab (39), sintilimab (40), camrelizumab (41), tislelizumab (42), penpulimab (43), prolgolimab (44), dostarlimab (45), and zimberelimab (46), and five PD-L1 inhibitors atezolizumab (47), durvalumab (48), avelumab (49), envafolimab (50), and sugemalimab (51) listed in succession. The Food and Drug Administration (FDA) has authorized six ICIs, whereas the National Medical Products Administration has approved 12 ICIs (NMPA) (52–55).

As the number of approved PD-1 and PD-L1 inhibitors continues to increase, each has a distinct therapeutic profile and clinical significance. This section provides more information on the specific characteristics of some of these inhibitors.

## 5 PD-1 inhibitors

### 5.1 Nivolumab (Opdivo)

Nivolumab is a human-derived monoclonal antibody belonging to the IgG4 class. It specifically binds to PD-1 present in T-cells. By inhibiting PD-1, nivolumab allows the immune system to recognize and target cancer cells more efficiently. The binding sites of nivolumab on PD-1 include the N-terminal extension, FG loop, and BC loop (56). Research indicates that the crystal structures of the PD-1 ectodomain, in conjunction with the Fab fragments of nivolumab, provide insight into the antibody’s precise epitopes and checkpoint inhibition mechanisms (57). There is also evidence suggesting a role for PD-1 glycosylation in nivolumab recognition (58).

Nivolumab is administered intravenously (IV) through an infusion. The frequency of these infusions can vary, but they are typically administered every 2 weeks or every 4 weeks (59). Nivolumab is commonly provided at 240 mg intravenously every two weeks or 480 mg intravenously every four weeks until disease progression (60).

This extensive range of uses demonstrates the transformational potential of nivolumab for cancer treatment. It has been approved as a monotherapy or in combination with other drugs for a variety of cancers, including classic Hodgkin lymphoma, non-small cell lung cancer, metastatic colorectal cancer, and advanced Merkel cell carcinoma (61–63). Additionally, in the notable Checkmate 037 trial, individuals with metastatic melanoma who had prior treatment with CTLA-4 received doses of 3 mg/kg nivolumab (64).

Nivolumab has shown promise in clinical studies, particularly when administered in combination therapy. An important study revealed that the combination of nivolumab and ipilimumab significantly increased the overall survival rate compared to conventional chemotherapy alone (63).

Despite its therapeutic advantages, nivolumab is not associated with adverse effects. Frequent side effects include nausea, digestive disturbances, mouth ulcers, changed taste sensations, skin manifestations, hormone abnormalities, liver problems, pain in hands or feet, fever, and broad body aches (65–68). In addition, identifying the correct dose of nivolumab for cancer immunotherapy remains difficult, providing both obstacles and learning opportunities (69).

### 5.2 Pembrolizumab (Keytruda)

Pembrolizumab is a humanized monoclonal antibody of the IgG4-kappa class specifically engineered to target the PD-1 receptor (70). Through its binding to PD-1, Pembrolizumab prevents certain cancer cells from inhibiting the immune system, thus promoting the effective eradication of these cells. Detailed high-resolution structural analysis has shown an intricate interaction between the pembrolizumab Fv fragment and the extracellular domain of human PD-1 (71). Furthermore, while the pembrolizumab epitope aligns more with the PD-L1 binding site than with nivolumab, their respective binding sites on PD-1 exhibit minimal overlap (72). By inhibiting the PD-1 pathway, pembrolizumab enhances the immune response against cancer cells (73).

Pembrolizumab is administered intravenously (IV) via an infusion pump over 30 min through an intravenous line that contains a sterile, non-pyrogenic, and low-protein binding instrument (74). It is indicated for conditions such as locally advanced or metastatic urothelial carcinoma in adults who are not eligible for cisplatin-containing chemotherapy. The dosage might be 200 mg IV every three weeks or 400 mg IV every six weeks (75).

The FDA approves Pembrolizumab for the treatment of several cancers, including melanoma-specific lung, head, neck, stomach, and breast variants. It is effective for metastatic melanoma, non-small cell lung cancer, and cervical cancer as a PD-1 blocking antibody. It also treats adult and pediatric patients with refractory primary mediastinal large B-cell lymphomas. In 2021, its combination with chemotherapy was approved for early-stage, high-risk, and triple-negative breast cancer. This agent targets tumor cell pathways to evade the immune system (76, 77).

Pembrolizumab, a revolutionary drug in the field of cancer therapeutics, has distinct advantages and disadvantages. One of its primary benefits is its ability to rejuvenate the immune system and enhance the capacity to identify and combat cancer cells. Furthermore, recent research underscores the potential of producing pembrolizumab in plants, wherein its structure remains consistent with the well-known brand Keytruda, suggesting alternative manufacturing avenues (78).

On the other hand, while pembrolizumab exhibits tremendous therapeutic potential, its administration is not without challenges. A substantial proportion (70%) of patients on this medication report immune-related adverse reactions, with common symptoms including fever, fatigue, cough, and disruptions in the digestive system. Moreover, dermatological complications such as maculopapular eruptions, pruritus, and hypopigmentation affect approximately 42% of those treated (79). Given these multifaceted implications, patients and physicians must weigh the benefits against the potential side effects when considering pembrolizumab treatment.

### 5.3 Cemiplimab (Libtayo)

Cemiplimab is a human monoclonal antibody belonging to the immunoglobulin G4 (IgG4) class. Cemiplimab specifically targets PD-1, which is frequently used by cancer cells to evade the immune response (80, 81). This selective binding to PD-1 obstructs its interaction with PD-L1, thus alleviating T-cell suppression. Intriguingly, while the heavy chain of cemiplimab primarily binds to PD-1, its light chain competes with PD-L1 binding to PD-1, a mechanism similar to that observed with camrelizumab, another PD-1-focused monoclonal antibody (82). Using recombinant technology, Cemiplimab was found to be a conserved region found in all human mAbs and a variable region that is specific to PD-1 (83).

Cemiplimab, marketed under the brand name LIBTAYO, was administered as an intravenous (IV) infusion. The recommended dosage is 350 mg administered over 30 min. This infusion is typically administered every 3 weeks by healthcare professionals (84, 85).

Cemiplimab is approved for treating advanced cutaneous squamous cell carcinoma (CSCC) and is under investigation for the treatment of non-small cell lung cancer (NSCLC) and recurrent basal cell carcinoma. Ongoing trials are also exploring its efficacy in other cancers, such as cervical and colorectal cancer. Use should be under expert guidance because of its potential side effects (86).

Cemiplimab specifically boosts the immune system’s ability to combat cancer. It has demonstrated effectiveness in treating advanced CSCC, especially in patients unsuitable for surgery or radiation (87). Additionally, cemiplimab provides an alternative for those who do not respond to traditional therapies, although it is crucial to weigh its benefits against potential side effects (88).

Treatment with Cemiplimab is not devoid of side effects. Patients often report symptoms such as fatigue, alopecia, peripheral neuropathy (tingling or burning sensations in the extremities), musculoskeletal pain, dermatological issues, gastrointestinal disturbances, and decreased appetite (89, 90). A pivotal caution is the potential of the drug to cross the placenta, suggesting that it might inflict harm on the developing fetus. Hence, its use is discouraged during pregnancy unless the anticipated therapeutic benefits notably surpass the potential fetal risks (91).

## 6 PD-L1 Inhibitors

### 6.1 Atezolizumab (Tecentriq)

Atezolizumab operates as a humanized monoclonal antibody that specializes in binding to the PD-L1 protein present in certain cancer cells. Atezolizumab belongs to the IgG1 isotype category and is a fully-humanized monoclonal antibody. This counteracts the ability of these cells to suppress the immune system, thereby inhibiting the checkpoint function of PD-L1. This action enables the immune system to target and attack malignant cells accurately. The molecule’s unique binding mechanism competes with PD-1 for the same surface region on PD-L1, thereby obstructing the PD-1 and PD-L1 interaction, as confirmed by crystallographic analysis (92–96).

Atezolizumab is infused intravenously. The regimen can vary, either every two, three, or four weeks, depending on the prescribed dosage. The duration of therapy is tailored to the type of cancer and the patient’s therapeutic response and is determined by the physician (97).

The FDA has given a nod to atezolizumab for the treatment of advanced urothelial carcinoma, which encapsulates certain types of bladder and urinary tract cancers. It is especially advocated for metastatic cases that cannot undergo surgical removal or specific medical scenarios. It can be used as a standalone treatment or in synergy with other drugs (98).

Atezolizumab can potentially reduce immunosuppressive signals by blocking PD-L1 protein. This action ensures that the immune system remains in an active state and is better poised to recognize and eliminate cancer cells. Another significant advantage of this method is its versatility. Atezolizumab can be harmoniously combined with other therapeutic agents to expand its efficacy across a spectrum of cancer types. This combinatorial approach amplifies its potential and provides patients with a comprehensive treatment strategy (94).

Although atezolizumab has promising therapeutic efficacy, it has potential side effects. The primary concern is their potential to induce immune-related liver conditions, some of which can be severe and even life-threatening. Common adverse reactions include fatigue, nausea, decreased appetite, and skin-related issues such as rashes. Some patients may also experience more severe side effects as a result of the immune system attacking healthy tissues and organs, which can lead to inflammation in vital organs such as the lungs, liver, and hormone-producing glands (99–102).

### 6.2 Durvalumab (Imfinzi)

Durvalumab functions as an immune checkpoint inhibitor. At the molecular level, durvalumab is categorized as an IgG1 monoclonal antibody used for human derivation. Specifically, it is a human-derived monoclonal antibody that targets the PD-L1 protein. Thus, durvalumab obstructs the suppression of the immune system by cancer cells, thereby bolstering the immune response against malignant cells. Crystallography of Durvalumab revealed its binding mode. It latches atop the PD-L1’s binding domain, effectively halting its interaction with PD-1. The antibody identified the protruding flexible loop of PD-L1 through a combination of hydrogen bonds, salt bridges, and van der Waals forces. Corresponding *in vitro* studies show that Durvalumab’s effectiveness in inhibiting PD-L1 activity correlates with its concentration (103–106).

Durvalumab is typically administered intravenously. The dosage was set at 10 mg/kg and administered twice a week. However, treatment duration can vary, ranging from two to four weeks, depending on disease progression and patient tolerance (107).

Durvalumab enhances the ability of the immune system to combat tumor cells (108). The FDA sanctioned durvalumab for the treatment of unresectable stage III non-small cell lung cancer (NSCLC), advanced urothelial carcinoma, small cell lung cancer (SCLC), and bladder cancer. Research exploring its efficacy in other cancer types is ongoing (109, 110).

In addition, durvalumab can be used to treat advanced urothelial carcinoma and other cancers. Owing to its superior safety profile and potential for combination therapy, durvalumab is a viable cancer therapeutic option. By targeting and suppressing the PD-L1 protein, it may be possible to reverse the inhibition of the immune system and strengthen its anti-cancer defenses (10).

Patients taking durvalumab should be monitored for adverse effects. There are grave concerns, including the possibility of severe liver damage that requires immediate medical intervention. Additionally, patients may experience allergic reactions, fatigue, skin rashes, digestive disturbances, and respiratory infections (111, 112).

### 6.3 Avelumab (Bavencio)

Avelumab acts by inhibiting PD-L1, a protein that certain cancer cells use to suppress the immune system. As a human IgG1 lambda monoclonal antibody, avelumab reinforces the ability of the immune system to identify and eliminate cancer cells. The intricate structure of avelumab allows it to use both its heavy chain (VH) and light chain (VL) regions to bind to the IgV domain of PD-L1. By preventing PD-L1 from connecting to its receptor, PD-1, on T-cells, avelumab ensures that the immune system remains active against malignant cells. The specific amino acid sequence and structural characteristics of an antibody are vital for its precision in recognizing and binding to PD-L1 (113–116).

Medical professionals administer avelumab intravenously, typically over an hour, with sessions recurring every two weeks. Notably, systemic exposure to the drug is directly proportional to the dose within the bi-weekly range of 3–20 mg/kg. Additionally, an approximately 1.3-fold systemic accumulation was observed when the drug was provided bi-weekly (117, 118).

Avelumab bolsters the ability of the immune system to combat tumor cells. Primarily approved for metastatic Merkel Cell Carcinoma (MCC), a rare skin cancer, its applications also span to advanced renal cell carcinoma (RCC) and bladder cancer. Avelumab is often combined with other medications, notably axitinib, as a frontline treatment for RCC. Its role in oncology is expanding, offering promise in the battle against these malignancies (119, 120).

One of the pivotal advantages of avelumab is its capacity to amplify the immune system response, facilitating robust defense against tumor cells. Avelumab has repeatedly demonstrated a positive safety profile in several clinical trials, reassuring healthcare professionals of its dependability. Given the drug’s applicability across several cancer types, its adaptability is also impressive, making it a useful asset in the field of oncology (121, 122).

Therefore, patients should be informed of the potential adverse effects of avelumab. These may include skin signs, such as redness, swelling, discomfort, or scaling, especially on the hands and feet. Other potential side effects include heart abnormalities, gastrointestinal pain, swelling of different body parts, chest tightness, and tingling sensations, particularly in the hands and feet (123–125).

## 7 Recent research trends in cancer immunotherapy

Recent studies have demonstrated the efficacy of PD-1/PD-L1 immune checkpoint inhibitors in the treatment of triple-negative metastatic breast cancer (126). ICI underscore the potential of these medicines to target cancer subgroups for which therapy choices have been restricted. Researchers are investigating the use of these medicines in conjunction with chemotherapy, radiation therapy, and other immunotherapies. Additionally, they are attempting to identify biomarkers that might predict which patients are most likely to benefit from these medicines. ICI may lead to more individualized treatment options and enhance treatment success (127, 128).

PD-1/PD-L1 inhibitors provide a more tailored approach to cancer treatment, which frequently results in better patient outcomes than conventional therapies. Using the body’s defense systems, these inhibitors are frequently associated with fewer adverse effects than conventional chemotherapy or radiation. The versatility of these therapies enables their use as independent treatments or in conjunction with other therapies for a wide range of cancer types (128).

However, despite the fact that PD-1/PD-L1 inhibitors have revolutionized cancer treatment, obstacles such as the development of resistance to these drugs continue to exist. The next section explores the mechanisms underlying this resistance and its therapeutic implications.

## 8 Resistance to PD-1 and PD-L1 inhibitors

The PD-1/PD-L1 blockade represents a significant advancement in cancer immunotherapy. Although PD-1/PD-L1 inhibitor therapy offers long-term and potentially curative clinical advantages, therapeutic resistance remains a significant obstacle to its widespread adoption. Approximately 20–30% of patients are predicted to respond favorably to PD-1/PD-L1 blocking treatment. However, not all patients respond to these inhibitors, and some initial responders resist treatment over time. Resistance to PD-1 and PD-L1 inhibitors can be either primary or acquired, and the underlying processes may be complex and overlap within a patient population (4). The PD-1/PD-L1 checkpoint inhibitor is generally well-tolerated, although the medication has common adverse reactions. Adverse effects include fatigue, intense skin itching (pruritus), loss of skin pigmentation (vitiligo), gastrointestinal disturbances (including diarrhea), inflammation of the colon (colitis), and reduction in appetite. Furthermore, cases of tuberculosis associated with this treatment have been reported (129).

Several potential resistance mechanisms have been proposed, including abnormal antigen expression, presentation, and identification. Second, inappropriate tumor-infiltrating lymphocytes (TILs), dysfunctional T-cells, and T-cell exhaustion. Third, the absence of class I major histocompatibility complex (MHC) expression. Fourth, the presence of immunosuppressive substances in the tumor’s microenvironment (Tregs, MDSCs, TAMs, IDO, VEGFA, and some immunosuppressive cytokines). Fifth is alternative immune checkpoint regulation (TIM-3, HHLA-2, VISTA, LAG-3, CTLA-4, Siglec-15, TIGIT, and BTLA). Sixth, genetic modifications, including mutations and gene amplification, are involved in antigen processing and presentation. Seventh are non-coding RNA (miRNAs and lncRNAs).In addition, the eighth factor - the microbiome’s impact on resistance and responsiveness to PD-1/PD-L1 blocking treatment–adds to this problem (129–132).

Emerging research offers hope by uncovering genetic mutations and the role of the gut microbiome in the resistance to PD-1/PD-L1 inhibitors (133). When combined with other treatments or alternating therapies, resistance may be mitigated. At the same time, these mechanisms of resistance present challenges in cancer therapy; ongoing research and innovative strategies offer promise for overcoming these barriers. The subsequent section will explore the potential approaches and methodologies to counteract and potentially reverse resistance to PD-1/PD-L1 inhibitors.

## 9 Overcome PD-1/PD-L1 inhibitor resistance

Several challenges must be addressed for PD-1/PD-L1 inhibitor therapy to have a significant clinical impact on a larger patient population. Several treatment techniques have been developed to overcome this resistance. One approach involves directing therapeutic interventions toward the tumor microenvironment to promote the infiltration of T-cells directly into the tumor site. The ability of cancer cells to exclude T lymphocytes from the tumor microenvironment appears to be an essential mechanism for resistance to anti-PD-1/PD-L1 treatment. Researchers are investigating combination treatments that promote T-cell infiltration, including chemotherapy, radiation therapy, and other immunotherapies (134, 135).

Another strategy is to target alternative immune checkpoints that may increase in response to PD-1/PD-L1 inhibition. The T-cell immunoglobulin and mucin domain 3 (TIM-3) pathway and lymphocyte activation gene 3 (LAG-3) pathway are among the potential targets for combination therapy (129). In conjunction with PD-1/PD-L1 inhibitor treatment, blocking the lactate/GPR81 pathway and administering metformin has been demonstrated to reduce tumor development and cause tumor regression (136).

In conjunction with PD-1/PD-L1 inhibitor treatment, blocking the lactate/GPR81 pathway and administering metformin has been proven to reduce tumor development and cause tumor regression. Combining the inhibition of these pathways may help overcome PD-1/PD-L1 inhibitor resistance (137, 138). According to one study, primary resistance to PD-1 inhibitors arises in immunosuppressive tumor settings caused by myeloid-derived suppressor cells (MDSCs) and T-cell exhaustion, increasing T regulatory cells (Tregs). Considering the activation of Tregs by TGF-β, the use of TGF-β inhibitors holds promise in overcoming the initial resistance to anti-PD-1 therapy. Recent studies involving mice have shown that the combined administration of anti-PD-1 and anti-TGF-β yields substantial therapeutic advantages compared with anti-TGF-β alone (139).

These findings underscore the significance of combination therapies for enhancing the efficacy of PD-1/PD-L1 inhibitors. As our understanding of resistance mechanisms deepens, the exploration of synergistic treatment combinations continues to gain traction, as discussed in the following section.

## 10 Current combination therapies with PD-1/PD-L1 inhibitors

The application of combination strategies that augment the immunogenicity of tumors has the potential to increase the effectiveness of PD-1/PD-L1 inhibition and other immune-oncology (IO) treatments. Regarding the clinical limits associated with anti-PD-1/PD-L1 monotherapy, combination therapies to address the mechanisms responsible for resistance to PD-1/PD-L1 inhibitors are becoming increasingly common. Ongoing research is investigating the combination of PD-1/PD-L1 inhibitors with small-molecule inhibitors that specifically target essential oncogenes or signaling molecules in various types of cancers. These include BRAF in melanoma, RET/PDGFR in renal cell carcinoma, VEGF-A in NSCLC, MEK in melanoma and NSCLC, and PI3K in multiple cancer types. As with chemotherapy, it is believed that the efficiency of specific small-molecule inhibitors is at least partially dependent on adaptive immune responses, and checkpoint inhibitors may increase these responses (140, 141).

Chemotherapy, VEGF/VEGFR-targeted treatments, and anti-CTLA-4 therapy are the most commonly used and effective therapies. Other options, such as radiotherapy, can complement PD-1/PD-L1 suppression. Existing data indicate that radiotherapy can modulate T-cell activity and boost PD-L1 expression, enhancing the efficacy of anti-PD-L1 treatment (142). In a preclinical study, the simultaneous administration of an anti-PD-1 blocking antibody, an anti-CD137 stimulating antibody, and vaccination therapy significantly enhanced T-cell activation in pancreatic ductal adenocarcinoma (143). Recent studies have investigated the use of PD-1/PD-L1 inhibitors as neoadjuvant therapy for surgically resectable tumors. In non-small cell lung cancer patients, neoadjuvant nivolumab treatment led to significant pathological responses in 9 of 21 surgically treated individuals. Despite the known toxicities of combining nivolumab and ipilimumab, two trials with small melanoma patient groups revealed promising outcomes using either neoadjuvant nivolumab alone or in combination with ipilimumab. In resectable glioblastoma, both pembrolizumab and nivolumab have shown potent immunological effects as neoadjuvant treatments. One study highlighted how neoadjuvant anti-PD-1 immunotherapy fortifies Tumor-Infiltrating Lymphocytes (TILs), inducing a pronounced interferon response within the tumor microenvironment. This activation leads to interferon-γ production by PD-1/PD-L1-suppressed T-cells, which are vital for priming tumor-specific T lymphocytes. The subsequent interferon surge halts tumor cell growth by downregulating cell cycle genes, offering a therapeutic advantage (129, 144).

Furthermore, marrying tumor resection with therapy magnifies the expansion of tumor-specific T-cells, while peripheral blood CD4+ T-cells exhibit an enhanced shift towards activation and memory, augmenting the post-surgical anti-tumor reaction. The study showed improved survival in patients administered pembrolizumab both pre-and post-surgery compared with those treated post-surgery alone. Recognizing their effectiveness, the FDA has greenlit both nivolumab and pembrolizumab for adjuvant treatment of surgically resectable melanoma (129, 144).

Recent clinical trial advancements with tiragolumab have garnered considerable attention in the oncology sphere. This agent, which targets TIGIT, shows marked efficacy in the treatment of metastatic non-small cell lung cancer (NSCLC), especially when paired with anti-PD-L1 therapies. Its promise earned it the “breakthrough therapy” designation by the FDA. The results from multiple studies have emphasized its therapeutic potential. The combined strategy of inhibiting both TIGIT and PD-L1 (such as atezolizumab) has shown heightened disruption of the defensive tactics of cancer cells. Ongoing investigations are exploring its efficacy across various cancer types, including small-cell lung cancer and esophageal cancer; this combination bolsters the immune response against tumors. As research progresses, the significance of nivolumab in oncology is increasingly highlighted, hinting at its broader application in cancer care (145).

Combining liposomes loaded with TNF-α and anti-PD-1/PD-L1 therapy boosted anti-tumor immune responses. The study carried out by Xia et al., 2021 utilized these liposomes to target tumors and induce necrosis, releasing tumor-specific antigens. This release boosts dendritic cell activation and T-cell infiltration. When paired with checkpoint blockade therapy, tumor cells effectively transform into endogenous vaccines, further enhancing the efficacy of anti-PD-1/PD-L1 therapy. Using neoantigens can augment the potency of immune checkpoint inhibitors (146).

## 11 Integration of PD-1/PD-L1 inhibitors with CAR T-cell therapy

The advent of Chimeric Antigen Receptor T (CAR T) cell therapy has considerably improved the field of cancer immunotherapy, particularly since its FDA clearance for the treatment of lymphoma. CAR T-cell therapy employs a patient’s T-cells to combat cancer by modifying them in the laboratory to seek out and kill cancer cells. Researchers are exploring combinational techniques using PD-1/PD-L1 inhibitors to further enhance anti-tumor responses in both hematological malignancies and solid tumors in light of the encouraging outcomes from CAR T-cell treatments (147, 148).

The goal of combining PD-1/PD-L1 inhibitors with CAR T-cell treatment is to combat the immunosuppressive tumor microenvironment that is frequently observed in cancer patients. The PD-1/PD-L1 inhibitors assist in removing the immune system’s brakes, enabling CAR T-cells to detect and destroy cancer cells. In addition, PD-1/PD-L1 inhibitors may aid in minimizing the fatigue phenotype frequently found in CAR T-cells when deployed against solid tumors, hence extending their effector capabilities. In preclinical animals, combining PD-1/PD-L1 inhibitors with CAR T cell treatment increased tumor regression and extended life. In recent research, it was shown that combining PD-1/PD-L1 inhibitors with CAR T-cell treatment enhanced the response rate and overall survival of patients with relapsed or resistant diffuse large B-cell lymphoma (DLBCL). Additionally, the study revealed that patients tolerated the combo treatment well (147–149). The interaction between PD-1/PD-L1 inhibitors and CAR T-cell treatment offers a promising technique for enhancing CAR T-cell effectiveness, potentially reducing the immunosuppressive tumor microenvironment and CAR T-cell fatigue, so opening the path for improved responses in a variety of cancers (148).

Future research will undoubtedly build on these positive clinical results. As combination therapies with PD-1/PD-L1 inhibitors continue to show promise, they have set the stage for a new era in cancer treatment. With the encouraging advancements made thus far, the next frontier lies in further understanding the long-term impact of these combinations and their role in shaping the future landscape of cancer immunotherapy.

## 12 Conclusion and future prospective

The advent of PD-1/PD-L1 therapies has been revolutionary in the field of cancer treatment. These inhibitors exploit the natural defenses of the immune system, specifically targeting the mechanisms by which tumors evade immune destruction. Their efficacy is evident across diverse cancer types, with results often surpassing those of conventional treatment. Significant outcomes, including prolonged life expectancy and curative responses to advanced diseases, underscore their potential.

While the promise of PD-1/PD-L1 therapies is undeniable, the field grapples with unresolved questions. Determining the optimal dosing, ensuring safety, pinpointing the exact efficacy, and overcoming both innate and acquired resistance pose significant challenges. Furthermore, understanding the intricacies of the mechanisms of these inhibitors underscores the need for more in-depth research.

The recognition of PD-1/PD-L1 inhibitors in clinical trials cements their role alongside established cancer treatments. However, challenges persist, such as patient identification for research and global competition in anti-PD-1/PD-L1 studies. Regulatory bodies such as the FDA stress the importance of fostering collaborative research, especially for combination therapies incorporating these inhibitors.

The future holds promise but also demands rigorous exploration. As the PD-1/PD-L1 drug landscape and associated clinical studies evolve, addressing challenges head-on becomes paramount. We envision that PD-1/PD-L1 blockade treatment will dominate the cancer immunotherapy domain in the coming years, with the hope that further insights into this signaling system will continue to illuminate and guide the field.

## Author contributions

AP: Conceptualization, Data curation, Visualization, Writing – original draft, Writing – review and editing. FC: Data curation, Writing – review and editing. PM: Data curation, Writing – review and editing. RK: Data curation, Writing – review and editing. KQ: Supervision, Validation, Visualization, Writing – review and editing. AA: Funding acquisition, Supervision, Validation, Writing – review and editing. HF: Conceptualization, Supervision, Validation, Visualization, Writing – review and editing.

## References

[1] UK CR. Worldwide cancer incidence statistics. Cancer Res UK (2023). http://www.cancerresearchuk.org/cancerinfo/cancerstats/world/incidence/ [Accessed September 10, 2023]

[2] Siegel RL Miller KD Wagle NS Jemal A. Cancer statistics, 2023. CA Cancer J Clin (2023) 73:17–48. doi: 10.3322/caac.21763 36633525

[3] Johdi NA Sukor NF. Colorectal cancer immunotherapy: options and strategies. Front Immunol (2020) 11:1624. doi: 10.3389/fimmu.2020.01624 33042104 PMC7530194

[4] Sun JY Zhang D Wu S Xu M Zhou X Lu XJ. Resistance to PD-1/PD-L1 blockade cancer immunotherapy: Mechanisms, predictive factors, and future perspectives. biomark Res (2020) 8:1–10. doi: 10.1186/s40364-020-00212-5 32864132 PMC7450549

[5] Payandeh Z Khalili S Somi MH Mard-Soltani M Baghbanzadeh A Hajiasgharzadeh K. PD-1/PD-L1-dependent immune response in colorectal cancer. J Cell Physiol (2020) 235:5461–75. doi: 10.1002/jcp.29494 31960962

[6] Ghosh C Luong G Sun Y. A snapshot of the PD-1/PD-L1 pathway. J Cancer (2021) 12:2735–46. doi: 10.7150/JCA.57334 PMC8040720 33854633

[7] Chen Y Pei Y Luo J Huang Z Yu J Meng X. Looking for the optimal PD-1/PD-L1 inhibitor in cancer treatment: A comparison in basic structure, function, and clinical practice. Front Immunol (2020) 11:1088. doi: 10.3389/fimmu.2020.01088 32547566 PMC7274131

[8] Jiang Y Zhao X Fu J Wang H. Progress and challenges in precise treatment of tumors with PD-1/PD-L1 blockade. Front Immunol (2020) 11:339. doi: 10.3389/fimmu.2020.00339 32226426 PMC7080697

[9] Wang B Bai W Ma H Li F. Regulatory effect of PD1/PD-ligand 1 (PD-L1) on treg cells in patients with idiopathic pulmonary fibrosis. Med Sci Monit (2020) 26:1–9. doi: 10.12659/MSM.927577 PMC7786833 33386384

[10] Ai L Chen J Yan H He Q Luo P Xu Z. Research status and outlook of pd-1/pd-l1 inhibitors for cancer therapy. Drug Des Devel Ther (2020) 14:3625–49. doi: 10.2147/DDDT.S267433 PMC7490077 32982171

[11] Munari E Mariotti FR Quatrini L Bertoglio P Tumino N Vacca P. PD-1/pd-l1 in cancer: Pathophysiological, diagnostic and therapeutic aspects. Int J Mol Sci (2021) 22:1–18. doi: 10.3390/ijms22105123 PMC8151504 34066087

[12] Han Y Liu D Li L. PD-1/PD-L1 pathway: current researches in cancer. Am J Cancer Res (2020) 10:727–42. PMC7136921 32266087

[13] Singh V Khurana A Allawadhi P Banothu AK Bharani KK Weiskirchen R. Emerging role of PD-1/PD-L1 inhibitors in chronic liver diseases. Front Pharmacol (2021) 12:790963. doi: 10.3389/fphar.2021.790963 35002724 PMC8733625

[14] Barrueto L Caminero F Cash L Makris C Lamichhane P Deshmukh RR. Resistance to checkpoint inhibition in cancer immunotherapy. Transl Oncol (2020) 13:100738. doi: 10.1016/j.tranon.2019.12.010 32114384 PMC7047187

[15] Yu X Gao R Li Y Zeng C. Regulation of PD-1 in T cells for cancer immunotherapy. Eur J Pharmacol (2020) 881:173240. doi: 10.1016/j.ejphar.2020.173240 32497624

[16] Liu X Yang L Tan X. PD-1/PD-L1 pathway: A double-edged sword in periodontitis. BioMed Pharmacother (2023) 159:114215. doi: 10.1016/j.biopha.2023.114215 36630848

[17] Ai L Xu A Xu J. Roles of PD-1/PD-L1 pathway: signaling, cancer, and beyond. Adv Exp Med Biol (2020) 1248:33–59. doi: 10.1007/978-981-15-3266-5_3 32185706

[18] Yi M Niu M Xu L Luo S Wu K. Regulation of PD-L1 expression in the tumor microenvironment. J Hematol Oncol (2021) 14:1–13. doi: 10.1186/s13045-020-01027-5 33413496 PMC7792099

[19] Kciuk M Kołat D Kałuzińska-Kołat Ż Gawrysiak M Drozda R Celik I. PD-1/PD-L1 and DNA damage response in cancer. Cells (2023) 12:1–31. doi: 10.3390/cells12040530 PMC9954559 36831197

[20] Salmaninejad A Valilou SF Shabgah AG Aslani S Alimardani M Pasdar A. PD-1/PD-L1 pathway: Basic biology and role in cancer immunotherapy. J Cell Physiol (2019) 234:16824–37. doi: 10.1002/jcp.28358 30784085

[21] Gou Q Dong C Xu H Khan B Jin J Liu Q. PD-L1 degradation pathway and immunotherapy for cancer. Cell Death Dis (2020) 11:955. doi: 10.1038/s41419-020-03140-2 33159034 PMC7648632

[22] Schönrich G Raftery MJ. The PD-1/PD-L1 axis and virus infections: A delicate balance. Front Cell Infect Microbiol (2019) 9:207. doi: 10.3389/fcimb.2019.00207 31263684 PMC6584848

[23] Xu X Masubuchi T Cai Q Zhao Y Hui E. Molecular features underlying differential SHP1/SHP2 binding of immune checkpoint receptors. Elife (2021) 10:1–25. doi: 10.7554/eLife.74276 PMC8631942 34734802

[24] Dermani FK Samadi P Rahmani G Kohlan AK Najafi R. PD-1/PD-L1 immune checkpoint: Potential target for cancer therapy. J Cell Physiol (2019) 234:1313–25. doi: 10.1002/jcp.27172 30191996

[25] Zhulai G Oleinik E. Targeting regulatory T cells in anti-PD-1/PD-L1 cancer immunotherapy. Scand J Immunol (2022) 95:1–15. doi: 10.1111/sji.13129 34936125

[26] Zhao SG Lehrer J Chang SL Das R Erho N Liu Y. The immune landscape of prostate cancer and nomination of PD-L2 as a potential therapeutic target. J Natl Cancer Inst (2019) 111:301–10. doi: 10.1093/jnci/djy141 30321406

[27] Xin Yu J Hodge JP Oliva C Neftelinov ST Hubbard-Lucey VM Tang J. Trends in clinical development for PD-1/PD-L1 inhibitors. Nat Rev Drug Discovery (2020) 19:163–4. doi: 10.1038/d41573-019-00182-w 32127660

[28] Sasikumar PG Ramachandra RK Adurthi S Dhudashiya AA Vadlamani S Vemula K. A rationally designed peptide antagonist of the PD-1 signaling pathway as an immunomodulatory agent for cancer therapy. Mol Cancer Ther (2019) 18:1081–91. doi: 10.1158/1535-7163.MCT-18-0737 31015307

[29] Cheng Y Sun F Cao H Gai D Peng B Xu H. NEK2 inhibition enhances the efficacy of PD-1/PD-L1 blockade in multiple myeloma. Blood (2021) 138:2671–1. doi: 10.1182/blood-2021-148659

[30] Islam MK Stanslas J. Peptide-based and small molecule PD-1 and PD-L1 pharmacological modulators in the treatment of cancer. Pharmacol Ther (2021) 227:107870. doi: 10.1016/j.pharmthera.2021.107870 33895183

[31] Yang J Hu L. Immunomodulators targeting the PD-1/PD-L1 protein-protein interaction: From antibodies to small molecules. Med Res Rev (2019) 39:265–301. doi: 10.1002/med.21530 30215856

[32] Liu J Chen Z Li Y Zhao W Wu JB Zhang Z. PD-1/PD-L1 checkpoint inhibitors in tumor immunotherapy. Front Pharmacol (2021) 12:731798. doi: 10.3389/fphar.2021.731798 34539412 PMC8440961

[33] AmeliMojarad M AmeliMojarad M Cui X. Prospective role of PD-1/PD-L1 immune checkpoint inhibitors in GI cancer. Pathol Res Pract (2023) 244:154338. doi: 10.1016/j.prp.2023.154338 36905697

[34] Sasikumar PG Ramachandra M. Small molecule agents targeting PD-1 checkpoint pathway for cancer immunotherapy: mechanisms of action and other considerations for their advanced development. Front Immunol (2022) 13:752065. doi: 10.3389/fimmu.2022.752065 35585982 PMC9108255

[35] Upadhaya S Neftelinov ST Hodge J Campbell J. Challenges and opportunities in the PD1/PDL1 inhibitor clinical trial landscape. Nat Rev Drug Discovery (2022) 21:482–3. doi: 10.1038/d41573-022-00030-4 35145263

[36] Wernham AGH Shah F Velangi S. Nivolumab PD-1 inhibitor immunotherapy associated with vulvar, perineal and perianal lichen sclerosus. Clin Exp Dermatol (2019) 44:e22–3. doi: 10.1111/ced.13825 30430637

[37] Barta SK Zain J MacFarlane AW Smith SM Ruan J Fung HC. Phase II study of the PD-1 inhibitor pembrolizumab for the treatment of relapsed or refractory mature T-cell lymphoma. Clin Lymphoma Myeloma Leuk (2019) 19:356–364.e3. doi: 10.1016/j.clml.2019.03.022 31029646 PMC7433797

[38] Davis CM Lewis KD. Brief overview: cemiplimab for the treatment of advanced basal cell carcinoma: PD-1 strikes again. Ther Adv Med Oncol (2022) 13:175883592110661. doi: 10.1177/17588359211066147 PMC8785268 35082923

[39] Jiao Y Liu M Luo N Guo H Li J. Successful treatment of advanced pulmonary sarcomatoid carcinoma with the PD-1 inhibitor toripalimab: A case report. Oral Oncol (2021) 112:104992. doi: 10.1016/j.oraloncology.2020.104992 32943323

[40] Gao S Li N Gao S Xue Q Ying J Wang S. Neoadjuvant PD-1 inhibitor (Sintilimab) in NSCLC. J Thorac Oncol (2020) 15:816–26. doi: 10.1016/j.jtho.2020.01.017 32036071

[41] Shen T Zheng S Geng L Liu Z Xu J Lin B. Experience with anti-PD-1 antibody, camrelizumab, monotherapy for biliary tract cancer patients and literature review. Technol Cancer Res Treat (2020) 19:153303382097970. doi: 10.1177/1533033820979703 PMC7739105 33308041

[42] Liu SY Wu YL. Tislelizumab: an investigational anti-PD-1 antibody for the treatment of advanced non-small cell lung cancer (NSCLC). Expert Opin Investig Drugs (2020) 29:1355–64. doi: 10.1080/13543784.2020.1833857 33044117

[43] Mislang ARA Coward J Cooper A Underhill CR Zheng Y Xu N. 157P Efficacy and safety of penpulimab (AK105), a new generation anti-programmed cell death-1 (PD-1) antibody, in upper gastrointestinal cancers. Ann Oncol (2020) 31:S1300–1. doi: 10.1016/j.annonc.2020.10.178

[44] Tjulandin S Demidov L Moiseyenko V Protsenko S Semiglazova T Odintsova S. Novel PD-1 inhibitor prolgolimab: expanding non-resectable/metastatic melanoma therapy choice. Eur J Cancer (2021) 149:222–32. doi: 10.1016/j.ejca.2021.02.030 33872982

[45] Cicala CM Musacchio L Scambia G Lorusso D. Dostarlimab: From preclinical investigation to drug approval and future directions. Hum Vaccines Immunother (2023) 19:217820. doi: 10.1080/21645515.2023.2178220 PMC10026921 36762991

[46] Lou B Wei H Yang F Wang S Yang B Zheng Y. Preclinical characterization of GLS-010 (Zimberelimab), a novel fully human anti-PD-1 therapeutic monoclonal antibody for cancer. Front Oncol (2021) 11:736955. doi: 10.3389/fonc.2021.736955 34604074 PMC8479189

[47] Rizzo A Ricci AD Brandi G. Atezolizumab in advanced hepatocellular carcinoma: Good things come to those who wait. Immunotherapy (2021) 13:637–44. doi: 10.2217/imt-2021-0026 33820447

[48] Rizzo A Ricci AD Brandi G. Durvalumab: an investigational anti-PD-L1 antibody for the treatment of biliary tract cancer. Expert Opin Investig Drugs (2021) 30:343–50. doi: 10.1080/13543784.2021.1897102 33645367

[49] Collins JM Gulley JL. Product review: avelumab, an anti-PD-L1 antibody. Hum Vaccines Immunother (2019) 15:891–908. doi: 10.1080/21645515.2018.1551671 PMC6605872 30481100

[50] Papadopoulos KP Harb W Peer CJ Hua Q Xu S Lu H. First-in-human phase I study of envafolimab, a novel subcutaneous single-domain anti-PD-L1 antibody, in patients with advanced solid tumors. Oncologist (2021) 26:e1514–25. doi: 10.1002/onco.13817 PMC8417852 33973293

[51] Sakamoto M Jimeno A. Sugemalimab, a novel PD-L1 inhibitor for treatment of advanced or metastatic non-small cell lung cancer. Drugs Today (2023) 59:169–77. doi: 10.1358/DOT.2023.59.3.3507759 36847625

[52] Awadasseid A Zhou Y Zhang K Tian K Wu Y Zhang W. Current studies and future promises of PD-1 signal inhibitors in cervical cancer therapy. BioMed Pharmacother (2023) 157:114057. doi: 10.1016/j.biopha.2022.114057 36463828

[53] Shang J Huang L Huang J Ren X Liu Y Feng Y. Population pharmacokinetic models of anti-PD-1 mAbs in patients with multiple tumor types: A systematic review. Front Immunol (2022) 13:871372. doi: 10.3389/fimmu.2022.871372 35983041 PMC9379304

[54] Sun Y Ma L Ma J Li B Zhu Y Chen F. Combined application of plant growth-promoting bacteria and iron oxide nanoparticles ameliorates the toxic effects of arsenic in Ajwain (Trachyspermum ammi L.). Front Plant Sci (2022) 13:1098755. doi: 10.3389/fpls.2022.1098755 36643291 PMC9832315

[55] Yan T Yu L Shangguan D Li W Liu N Chen Y. Advances in pharmacokinetics and pharmacodynamics of PD-1/PD-L1 inhibitors. Int Immunopharmacol (2023) 115:109638. doi: 10.1016/j.intimp.2022.109638 36587500

[56] Guo L Wei R Lin Y Kwok HF. Clinical and recent patents applications of PD-1/PD-L1 targeting immunotherapy in cancer treatment—Current progress, strategy, and future perspective. Front Immunol (2020) 11:1508. doi: 10.3389/fimmu.2020.01508 32733486 PMC7358377

[57] Jeong TJ Lee HT Gu N Jang YJ Choi SB Park UB. The high-resolution structure reveals remarkable similarity in PD-1 binding of cemiplimab and dostarlimab, the FDA-approved antibodies for cancer immunotherapy. Biomedicines (2022) 10:3154. doi: 10.3390/biomedicines10123154 36551910 PMC9775377

[58] Wu Q Jiang L cheng L jun H Yang B Cao J. Small molecule inhibitors targeting the PD-1/PD-L1 signaling pathway. Acta Pharmacol Sin (2021) 42:1–9. doi: 10.1038/s41401-020-0366-x PMC7921448 32152439

[59] bristol-Myers Squibb. Getting an infusion | Previously treated advanced NSCLC | OPDIVO® (nivolumab) (2016). Available at: http://www.opdivo.bmscustomerconnect.com/advanced-nsclc/getting-an-infusion.

[60] Opdivo (nivolumab) dosing, indications, interactions, adverse effects, and more (2022). Available at: https://reference.medscape.com/drug/opdivo-nivolumab-999989.

[61] André T Lonardi S Wong KYM Lenz HJ Gelsomino F Aglietta M. Nivolumab plus low-dose ipilimumab in previously treated patients with microsatellite instability-high/mismatch repair-deficient metastatic colorectal cancer: 4-year follow-up from CheckMate 142. Ann Oncol (2022) 33:1052–60. doi: 10.1016/j.annonc.2022.06.008 35764271

[62] Luo Y Sun N He J. Nivolumab plus ipilimumab: a potential regimen to rewrite treatment guidelines for ESCC. Signal Transduct Target Ther (2022) 7:169. doi: 10.1038/s41392-022-01022-x 35614047 PMC9132968

[63] Baas P Scherpereel A Nowak AK Fujimoto N Peters S Tsao AS. First-line nivolumab plus ipilimumab in unresectable Malignant pleural mesothelioma (CheckMate 743): a multicentre, randomised, open-label, phase 3 trial. Lancet (2021) 397:375–86. doi: 10.1016/S0140-6736(20)32714-8 33485464

[64] Smylie MG. Use of immuno-oncology in melanoma. Curr Oncol (2020) 27:51–8. doi: 10.3747/co.27.5135 PMC7194004 32368174

[65] Janjigian YY Shitara K Moehler M Garrido M Salman P Shen L. First-line nivolumab plus chemotherapy versus chemotherapy alone for advanced gastric, gastro-oesophageal junction, and oesophageal adenocarcinoma (CheckMate 649): a randomised, open-label, phase 3 trial. Lancet (2021) 398:27–40. doi: 10.1016/S0140-6736(21)00797-2 34102137 PMC8436782

[66] Patel C Purkey SC Stancher J. S2958 acute appendicitis: A rare complication from nivolumab. Am J Gastroenterol (2021) 116:S1224–5. doi: 10.14309/01.ajg.0000785364.58385.99

[67] Almutairi AR McBride A Slack M Erstad BL Abraham I. Potential immune-related adverse events associated with monotherapy and combination therapy of ipilimumab, nivolumab, and pembrolizumab for advanced melanoma: A systematic review and meta-analysis. Front Oncol (2020) 10:91. doi: 10.3389/fonc.2020.00091 32117745 PMC7033582

[68] Julien K Leung HT Fuertes C Mori M Wang M-J Teo J. Nivolumab in advanced hepatocellular carcinoma: safety profile and select treatment-related adverse events from the checkMate 040 study. Oncologist (2020) 25:e1532–40. doi: 10.1634/theoncologist.2019-0591 PMC7543234 33400305

[69] Zhao X Shen J Ivaturi V Gopalakrishnan M Feng Y Schmidt BJ. Model-based evaluation of the efficacy and safety of nivolumab once every 4 weeks across multiple tumor types. Ann Oncol (2020) 31:302–9. doi: 10.1016/j.annonc.2019.10.015 31959348

[70] Ramos Perez J Montalban-Bravo G. Emerging drugs for the treatment of chronic myelomonocytic leukemia. Expert Opin Emerg Drugs (2020) 25:515–29. doi: 10.1080/14728214.2020.1854224 33280448

[71] Nomura N Nomura Y Sato Y Iwata S. Crystallographic approaches to study the interaction modes of PD-1- and CTLA-4-blocking antibodies. Methods Enzymology (2019) 629:383–99. doi: 10.1016/bs.mie.2019.10.008 31727250

[72] Routh ED Woodcock MG Beckabir W Vensko SP Serody JS Vincent BG. Evaluation of tumor antigen-specific antibody responses in patients with metastatic triple negative breast cancer treated with cyclophosphamide and pembrolizumab. J Immunother Cancer (2023) 11:1–18. doi: 10.1136/jitc-2022-005848 36882226 PMC10008414

[73] Li TR Chatterjee M Lala M Abraham AK Freshwater T Jain L. Pivotal dose of pembrolizumab: A dose-finding strategy for immuno-oncology. Clin Pharmacol Ther (2021) 110:200–9. doi: 10.1002/cpt.2170 33462831

[74] Preparation, storage and administration of KEYTRUDA® (pembrolizumab). IHCP. https://www.keytrudahcp.com/dosing/preparation-storageadministration/ [Accessed September 10, 2023]

[75] Keytruda (pembrolizumab) dosing, indications, interactions, adverse effects, and more. https://reference.medscape.co m/drug/keytrudapembrolizumab- 999962 [Accessed September 10, 2023]

[76] Schmid P Cortes J Pusztai L McArthur H Kümmel S Bergh J. Pembrolizumab for early triple-negative breast cancer. N Engl J Med (2020) 382:810–21. doi: 10.1056/nejmoa1910549 32101663

[77] Bagegni NA Davis AA Clifton KK Ademuyiwa FO. Targeted treatment for high-risk early-stage triple-negative breast cancer: spotlight on pembrolizumab. Breast Cancer Targets Ther (2022) 14:113–23. doi: 10.2147/BCTT.S293597 PMC9064451 35515356

[78] Phakham T Bulaon CJI Khorattanakulchai N Shanmugaraj B Buranapraditkun S Boonkrai C. Functional characterization of pembrolizumab produced in nicotiana benthamiana using a rapid transient expression system. Front Plant Sci (2021) 12:736299. doi: 10.3389/fpls.2021.736299 34567049 PMC8459022

[79] Shalata W Weissmann S Itzhaki Gabay S Sheva K Abu Saleh O Jama AA. A retrospective, single-institution experience of bullous pemphigoid as an adverse effect of immune checkpoint inhibitors. Cancers (Basel) (2022) 14:5451. doi: 10.3390/cancers14215451 36358869 PMC9656349

[80] Burova E Hermann A Dai J Ullman E Halasz G Potocky T. Preclinical development of the anti-LAG-3 antibody REGN3767: Characterization and activity in combination with the anti-PD-1 antibody cemiplimab in human PD-1xLAG-3–knockin mice. Mol Cancer Ther (2019) 18:2051–62. doi: 10.1158/1535-7163.MCT-18-1376 31395688

[81] Nguyen JH Epling D Dolphin N Paccaly A Conrado D Davis JD. Population pharmacokinetics modeling and exposure-response analyses of cemiplimab in patients with recurrent or metastatic cervical cancer. CPT Pharmacometrics Syst Pharmacol (2022) 11:1458–71. doi: 10.1002/psp4.12855 PMC9662200 36251220

[82] Liu K Tan S Jin W Guan J Wang Q Sun H. N-glycosylation of PD-1 promotes binding of camrelizumab. EMBO Rep (2020) 21:e51444. doi: 10.15252/embr.202051444 33063473 PMC7726772

[83] Posner J Barrington P Brier T Datta-Mannan A. Monoclonal antibodies: Past, present and future. In: Handbook of Experimental Pharmacology (Springer)(2019). p. 81–141. doi: 10.1007/164_2019_323 31820172

[84] Dosing and administration for the NINLARO® (ixazomib) regimen . Available at: https://www.ninlarohcp.com/dosing-administration.

[85] Lee A Duggan S Deeks ED. Cemiplimab: A review in advanced cutaneous squamous cell carcinoma. Drugs (2020) 80:813–9. doi: 10.1007/s40265-020-01302-2 32306208

[86] Zucali PA Lin CC Carthon BC Bauer TM Tucci M Italiano A. Targeting CD38 and PD-1 with isatuximab plus cemiplimab in patients with advanced solid Malignancies: Results from a phase I/II open-label, multicenter study. J Immunother Cancer (2022) 10:e003697. doi: 10.1136/jitc-2021-003697 35058326 PMC8783811

[87] Mager L Gardeen S Carr DR Shahwan KT. Cemiplimab for the treatment of advanced cutaneous squamous cell carcinoma: appropriate patient selection and perspectives. Clin Cosmet Investig Dermatol (2023) 16:2135–42. doi: 10.2147/CCID.S381471 PMC10423569 37581012

[88] Ríos-Viñuela E Álvarez P Lavernia J Serra-Guillén C Requena C Bernia E. Cemiplimab in advanced cutaneous squamous cell carcinoma: real-world experience in a monographic oncology center. Actas Dermosifiliogr (2022) 113:T610–5. doi: 10.1016/j.ad.2022.05.001 35431057

[89] Akhtar K Sravanthi MV D’Angelo J Sivapiragasam A. Cemiplimab for locally advanced cutaneous squamous cell carcinoma: A case series of 3 unique scenarios. J Investig Med High Impact Case Rep (2022) 10:232470962211214. doi: 10.1177/23247096221121408 PMC9421057 36017984

[90] Dumann K Artz N Ziemer M. Complete Remission of Basal Cell Carcinoma following Treatment with Cemiplimab after 2 Years. JAMA Dermatol (2021) 157:1004–6. doi: 10.1001/jamadermatol.2021.2206 34232273

[91] Damsin T Lebas E Marchal N Rorive A Nikkels AF. Cemiplimab for locally advanced and metastatic basal cell carcinoma. Expert Rev Anticancer Ther (2022) 22:243–8. doi: 10.1080/14737140.2022.2043748 35175882

[92] Gonzalez Martin A Sanchez Lorenzo L Colombo N Depont Christensen R Heitz F Meirovitz M. A phase III, randomized, double blinded trial of platinum based chemotherapy with or without atezolizumab followed by niraparib maintenance with or without atezolizumab in patients with recurrent ovarian, tubal, or peritoneal cancer and platinum treatment. Int J Gynecol Cancer (2021) 31:617–22. doi: 10.1136/ijgc-2020-001633 33318079

[93] Phetphoung T Malla A Rattanapisit K Pisuttinusart N Damrongyot N Joyjamras K. Expression of plant-produced anti-PD-L1 antibody with anoikis sensitizing activity in human lung cancer cells via., suppression on epithelial-mesenchymal transition. PloS One (2022) 17:e0274737. doi: 10.1371/journal.pone.0274737 36367857 PMC9651560

[94] Ding D Hu H Liao M Shi Y She L Yao L. Cost-effectiveness analysis of atezolizumab plus chemotherapy in the first-line treatment of metastatic non-squamous non-small cell lung cancer. Adv Ther (2020) 37:2116–26. doi: 10.1007/s12325-020-01292-3 32193809

[95] Guo X Sheng X. Drug discovery of PD-L1 inhibitor Atezolizumab. Highlights Sci Eng Technol (2022) 8:660–7. doi: 10.54097/hset.v8i.1253

[96] Li M Zhao R Chen J Tian W Xia C Liu X. Next generation of anti-PD-L1 Atezolizumab with enhanced anti-tumor efficacy in *vivo* . Sci Rep (2021) 11:5774. doi: 10.1038/s41598-021-85329-9 33707569 PMC7952408

[97] Røssevold AH Andresen NK Bjerre CA Gilje B Jakobsen EH Raj SX. Atezolizumab plus anthracycline-based chemotherapy in metastatic triple-negative breast cancer: the randomized, double-blind phase 2b ALICE trial. Nat Med (2022) 28:2573–83. doi: 10.1038/s41591-022-02126-1 PMC9800277 36482103

[98] Sternberg CN Loriot Y James N Choy E Castellano D Lopez-Rios F. a multinational single-arm safety study of atezolizumab therapy for locally advanced or metastatic urothelial or nonurothelial carcinoma of the urinary tract. Eur Urol (2019) 76:73–81. doi: 10.1016/j.eururo.2019.03.015 30910346

[99] Honma Y Shibata M Gohda T Matsumiya H Kumamoto K Miyama A. Rapid progression of liver fibrosis induced by acute liver injury due to immune-related adverse events of atezolizumab. Intern Med (2021) 60:1847–53. doi: 10.2169/internalmedicine.6535-20 PMC8263175 33456046

[100] Tie Y Yang H Zhao R Zheng H Yang D Zhao J. Safety and efficacy of atezolizumab in the treatment of cancers: A systematic review and pooled-analysis. Drug Des Devel Ther (2019) 13:523–38. doi: 10.2147/DDDT.S188893 PMC6366347 30787594

[101] Fa’ak F Vanegas D Osei KM. A case report of atezolizumab induced tumor lysis syndrome. Am J Case Rep (2019) 20:785–9. doi: 10.12659/AJCR.915351 PMC6561135 31160547

[102] Acikgoz O Bayramgil A Cavusoglu G Sadri S. Rare side effect caused by atezolizumab, an immune checkpoint inhibitor: Cold agglutinin disease. J Oncol Pharm Pract (2021) 27:2066–8. doi: 10.1177/10781552211033009 34282980

[103] Alvarez-Argote J Dasanu CA. Durvalumab in cancer medicine: a comprehensive review. Expert Opin Biol Ther (2019) 19:927–35. doi: 10.1080/14712598.2019.1635115 31272242

[104] Varlotto JM Sun Z Ramalingam SS Wakelee HA Lovly CM Oettel KR. Randomized phase III Trial of MEDI4736 (durvalumab) as concurrent and consolidative therapy or consolidative therapy alone for unresectable stage 3 NSCLC: A trial of the ECOG-ACRIN Cancer Research Group (EA5181). J Clin Oncol (2021) 39:TPS8584–TPS8584. doi: 10.1200/jco.2021.39.15_suppl.tps8584

[105] Goudy OJ Peng A Tripathy A Kuhlman B. Design of a protease-activated PD-L1 inhibitor. Protein Sci (2023) 32:e4578. doi: 10.1002/pro.4578 36705186 PMC9926466

[106] Lee HT Lee SH Heo YS. Molecular interactions of antibody drugs targeting PD-1, PD-L1, and CTLA-4 in immuno-oncology. Molecules (2019) 24:1190. doi: 10.3390/molecules24061190 30917623 PMC6470598

[107] Avrillon V Daniel C Boisselier P Le Péchoux C Chouaid C. Nationwide real-life safety and treatment exposure data on durvalumab after concurrent chemoradiotherapy in unresectable stage III, locally advanced, non-small cell lung cancer: analysis of patients enrolled in the french early access program. Lung (2022) 200:95–105. doi: 10.1007/s00408-022-00511-8 35141799

[108] Shiravand Y Khodadadi F Kashani SMA Hosseini-Fard SR Hosseini S Sadeghirad H. Immune checkpoint inhibitors in cancer therapy. Curr Oncol (2022) 29:3044–60. doi: 10.3390/curroncol29050247 PMC9139602 35621637

[109] Kaur J Elms J Munn AL Good D Wei MQ. Immunotherapy for non-small cell lung cancer (NSCLC), as a stand-alone and in combination therapy. Crit Rev Oncol Hematol (2021) 164:103417. doi: 10.1016/j.critrevonc.2021.103417 34242772

[110] Garcia del Muro X Valderrama BP Medina A Cuellar MA Etxaniz O Gironés Sarrió R. Phase II trial of durvalumab plus tremelimumab with concurrent radiotherapy (RT) in patients (pts) with localized muscle invasive bladder cancer (MIBC) treated with a selective bladder preservation approach: IMMUNOPRESERVE-SOGUG trial. J Clin Oncol (2021) 39:4505–5. doi: 10.1200/jco.2021.39.15_suppl.4505

[111] Shah P Sundaram V Björnsson E. Biologic and checkpoint inhibitor-induced liver injury: A systematic literature review. Hepatol Commun (2020) 4:172–84. doi: 10.1002/hep4.1465 PMC6996412 32025603

[112] Zhou Q Zhao J Wang J Bao G Gong LY. Durvalumab monotherapy as a third-line treatment for extensive-stage small-cell lung cancer: A case report. Ann Cardiothorac Surg (2020) 9:2386–92. doi: 10.21037/apm-20-1244 32692233

[113] Dudzisz-Śledź M Zwierzchowska M Bylina E Rutkowski P Czarnecka AM. Avelumab use in Merkel cell carcinoma treatment. Nowotwory (2022) 72:365–71. doi: 10.5603/NJO.a2022.0048

[114] Roviello G D’Angelo A Generali D Pittacolo M Ganzinelli M Iezzi G. Avelumab in gastric cancer. Immunotherapy (2019) 11:759–68. doi: 10.2217/imt-2019-0011 31060469

[115] Liu W Chen T Lai S Zhang G Liu G Jin H. (2021). Identifying the key residues regulating the binding between antibody avelumab and PD-L1 VIA molecular dynamics simulation, in: ACM Int Conf Proceeding Ser, New York, NY, USA. pp. 557–61. doi: 10.1145/3457682.3457767

[116] Lin X Lu X Luo G Xiang H. Progress in PD-1/PD-L1 pathway inhibitors: From biomacromolecules to small molecules. Eur J Med Chem (2020) 186:111876. doi: 10.1016/j.ejmech.2019.111876 31761384

[117] Wood LS Conway D Lapuente M Salvador G Fernandez Gomez S Carroll Bullock A. Avelumab first-line maintenance treatment in advanced bladder cancer: practical implementation steps for infusion nurses. J Infus Nurs (2022) 45:142–53. doi: 10.1097/NAN.0000000000000465 PMC9071022 35537002

[118] Loeb DM Lee JW Morgenstern DA Samson Y Uyttebroeck A Lyu CJ. Avelumab in paediatric patients with refractory or relapsed solid tumours: dose-escalation results from an open-label, single-arm, phase 1/2 trial. Cancer Immunol Immunother (2022) 71:2485–95. doi: 10.1007/s00262-022-03159-8 PMC9463244 35262780

[119] Bhatia S Nghiem P Veeranki SP Vanegas A Lachance K Tachiki L. Real-world clinical outcomes with avelumab in patients with Merkel cell carcinoma treated in the USA: A multicenter chart review study. J Immunother Cancer (2022) 10:e004904. doi: 10.1136/jitc-2022-004904 35981787 PMC9394192

[120] Hamilton G. Avelumab: search for combinations of immune checkpoint inhibition with chemotherapy. Expert Opin Biol Ther (2021) 21:311–22. doi: 10.1080/14712598.2021.1825679 32954871

[121] Patel MR Ellerton J Infante JR Agrawal M Gordon M Aljumaily R. Avelumab in metastatic urothelial carcinoma after platinum failure (JAVELIN Solid Tumor): pooled results from two expansion cohorts of an open-label, phase 1 trial. Lancet Oncol (2018) 19:51–64. doi: 10.1016/S1470-2045(17)30900-2 29217288 PMC7984727

[122] Kaczmarek M Poznańska J Fechner F Michalska N Paszkowska S Napierała A. Cancer vaccine therapeutics: limitations and effectiveness—A literature review. Cells (2023) 12:2159. doi: 10.3390/cells12172159 37681891 PMC10486481

[123] Zhou YW Xu Q Wang Y Xia RL Liu JY Ma XL. Immune checkpoint inhibitor-associated ophthalmic adverse events: current understanding of its mechanisms, diagnosis, and management. Int J Ophthalmol (2022) 15:646–56. doi: 10.18240/ijo.2022.04.19 PMC8995716 35450191

[124] Bolze PA You B Lotz JP Massardier J Gladieff L Joly F. Successful pregnancy in a cancer patient previously cured of a gestational trophoblastic tumor by immunotherapy. Ann Oncol (2020) 31:823–5. doi: 10.1016/j.annonc.2020.02.015 32171750

[125] Powles T Park SH Voog E Caserta C Valderrama BP Gurney H. Plain language summary of results from the JAVELIN Bladder 100 study: Avelumab maintenance treatment for advanced urothelial cancer. Futur Oncol (2022) 18:2361–71. doi: 10.2217/fon-2021-1631 35416053

[126] Yu Y Jin X Zhu X Xu Y Si W Zhao J. PD-1/PD-L1 immune checkpoint inhibitors in metastatic triple-negative breast cancer: a systematic review and meta-analysis. Front Immunol (2023) 14:1206689. doi: 10.3389/fimmu.2023.1206689 37377959 PMC10292799

[127] Sankar K Ye JC Li Z Zheng L Song W Hu-Lieskovan S. The role of biomarkers in personalized immunotherapy. biomark Res (2022) 10:32. doi: 10.1186/s40364-022-00378-0 35585623 PMC9118650

[128] Zhang Y Zhang Z. The history and advances in cancer immunotherapy: understanding the characteristics of tumor-infiltrating immune cells and their therapeutic implications. Cell Mol Immunol (2020) 17:807–21. doi: 10.1038/s41423-020-0488-6 PMC7395159 32612154

[129] Shergold AL Millar R Nibbs RJB. Understanding and overcoming the resistance of cancer to PD-1/PD-L1 blockade. Pharmacol Res (2019) 145:104258. doi: 10.1016/j.phrs.2019.104258 31063806

[130] Jaccard A Ho PC. The hidden side of PD-L1. Nat Cell Biol (2020) 22:1031–2. doi: 10.1038/s41556-020-0568-y 32839550

[131] Yuan Y Adam A Zhao C Chen H. Recent advancements in the mechanisms underlying resistance to pd-1/pd-l1 blockade immunotherapy. Cancers (Basel) (2021) 13:1–18. doi: 10.3390/cancers13040663 PMC7915065 33562324

[132] Tanoue T Morita S Plichta DR Skelly AN Suda W Sugiura Y. A defined commensal consortium elicits CD8 T cells and anti-cancer immunity. Nature (2019) 565:600–5. doi: 10.1038/s41586-019-0878-z 30675064

[133] Rebersek M. Gut microbiome and its role in colorectal cancer. BMC Cancer (2021) 21:1325. doi: 10.1186/s12885-021-09054-2 34895176 PMC8666072

[134] Oba T Long MD Keler T Marsh HC Minderman H Abrams SI. Overcoming primary and acquired resistance to anti-PD-L1 therapy by induction and activation of tumor-residing cDC1s. Nat Commun (2020) 11:5415. doi: 10.1038/s41467-020-19192-z 33110069 PMC7592056

[135] Yamaguchi H Hsu JM Yang WH Hung MC. Mechanisms regulating PD-L1 expression in cancers and associated opportunities for novel small-molecule therapeutics. Nat Rev Clin Oncol (2022) 19:287–305. doi: 10.1038/s41571-022-00601-9 35132224

[136] Kim Y Vagia E Viveiros P Kang CY Lee JY Gim G. Overcoming acquired resistance to PD-1 inhibitor with the addition of metformin in small cell lung cancer (SCLC). Cancer Immunol Immunother (2021) 70:961–5. doi: 10.1007/s00262-020-02703-8 PMC10991863 33084943

[137] Shen Q Reedijk M. Notch signaling and the breast cancer microenvironment. Adv Exp Med Biol (2021) 1287:183–200. doi: 10.1007/978-3-030-55031-8_12 33034033

[138] Shi W Lv L Liu N Wang H Wang Y Zhu W. A novel anti-PD-L1/IL-15 immunocytokine overcomes resistance to PD-L1 blockade and elicits potent antitumor immunity. Mol Ther (2023) 31:66–77. doi: 10.1016/j.ymthe.2022.08.016 36045584 PMC9840182

[139] Siewe N Friedman A. TGF-β inhibition can overcome cancer primary resistance to PD-1 blockade: A mathematical model. PloS One (2021) 16:e0252620. doi: 10.1371/journal.pone.0252620 34061898 PMC8168900

[140] Reck M Mok TS Nishio M Jotte RM Cappuzzo F Orlandi F. Atezolizumab plus bevacizumab and chemotherapy in non-small-cell lung cancer (IMpower150): key subgroup analyses of patients with EGFR mutations or baseline liver metastases in a randomised, open-label phase 3 trial. Lancet Respir Med (2019) 7:387–401. doi: 10.1016/S2213-2600(19)30084-0 30922878

[141] Prestipino A Zeiser R. Clinical implications of tumor-intrinsic mechanisms regulating PD-L1. Sci Transl Med (2019) 11:eaav4810. doi: 10.1126/scitranslmed.aav4810 30728286

[142] Chen C Liu Y Cui B. Effect of radiotherapy on T cell and PD-1 / PD-L1 blocking therapy in tumor microenvironment. Hum Vaccines Immunother (2021) 17:1555–67. doi: 10.1080/21645515.2020.1840254 PMC8117671 33428533

[143] Muth ST Saung MT Blair AB Henderson MG Thomas DL Zheng L. CD137 agonist-based combination immunotherapy enhances activated, effector memory T cells and prolongs survival in pancreatic adenocarcinoma. Cancer Lett (2021) 499:99–108. doi: 10.1016/j.canlet.2020.11.041 33271264 PMC7779747

[144] Cloughesy TF Mochizuki AY Orpilla JR Hugo W Lee AH Davidson TB. Neoadjuvant anti-PD-1 immunotherapy promotes a survival benefit with intratumoral and systemic immune responses in recurrent glioblastoma. Nat Med (2019) 25:477–86. doi: 10.1038/s41591-018-0337-7 PMC6408961 30742122

[145] American Association for Cancer Research.Tiragolumab impresses in multiple trials. Cancer Discovery (2020) 10:1086–7. doi: 10.1158/2159-8290.CD-NB2020-063 32576590

[146] Xia GQ Lei TR Yu TB Zhou PH. Nanocarrier-based activation of necroptotic cell death potentiates cancer immunotherapy. Nanoscale (2021) 13:1220–30. doi: 10.1039/d0nr05832g 33404038

[147] Yi M Zheng X Niu M Zhu S Ge H Wu K. Combination strategies with PD-1/PD-L1 blockade: current advances and future directions. Mol Cancer (2022) 21:28. doi: 10.1186/s12943-021-01489-2 35062949 PMC8780712

[148] Zhou Y Mu W Wang C Zhuo Z Xin Y Li H. Ray of dawn: Anti-PD-1 immunotherapy enhances the chimeric antigen receptor T-cell therapy in Lymphoma patients. BMC Cancer (2023) 23:1019. doi: 10.1186/s12885-023-11536-4 37872514 PMC10591343

[149] American Association for Cancer Research.Augmenting CAR T cells with PD-1 blockade. Cancer Discovery (2019) 9:158. doi: 10.1158/2159-8290.CD-NB2018-165 30523001

